# Strong Environmental and Genome‐Wide Population Differentiation Underpins Adaptation and High Genomic Vulnerability in the Dominant Australian Kelp (*Ecklonia radiata*)

**DOI:** 10.1002/ece3.71158

**Published:** 2025-05-12

**Authors:** Antoine J. P. Minne, Sofie Vranken, David Wheeler, Georgina Wood, Jacqueline Batley, Thomas Wernberg, Melinda A. Coleman

**Affiliations:** ^1^ UWA Oceans Institute Crawley Western Australia Australia; ^2^ School of Biological Sciences University of Western Australia Crawley Western Australia Australia; ^3^ Biology Department, Research Group Phycology Ghent University Ghent Belgium; ^4^ New South Wales Department of Primary Industries Orange Agricultural Institute Orange New South Wales Australia; ^5^ Flinders University Adelaide South Australia Australia; ^6^ Institute of Marine Research His Norway; ^7^ New South Wales Fisheries National Marine Science Centre Coffs Harbour New South Wales Australia; ^8^ National Marine Science Centre Southern Cross University Coffs Harbour New South Wales Australia

**Keywords:** climate change, genotype–environment association, latitudinal gradient, population genetics

## Abstract

Ongoing and predicted range loss of kelp forests in response to climatic stressors is pressing marine managers to look into the adaptive capacity of populations to inform conservation strategies. Characterising how adaptive genetic diversity and structure relate to present and future environmental variation represents an emerging approach to quantifying kelp vulnerability to environmental change and identifying populations with genotypes that potentially confer an adaptive advantage in future ocean conditions. The dominant Australian kelp, *Ecklonia radiata*, was genotyped from 10 locations spanning 2000 km of coastline and a 9.5°C average temperature gradient along the east coast of Australia, a global warming hotspot. ddRAD sequencing generated 10,700 high‐quality single nucleotide polymorphisms (SNPs) and characterized levels of neutral and adaptive genomic diversity and structure. The adaptive dataset, reflecting portions of the genome putatively under selection, was used to infer genomic vulnerability by 2050 under the RCP 8.5 scenario. There was strong neutral genetic differentiation between Australia mainland and Tasmanian populations, but only weak genetic structure among mainland populations within the main path of the East Australian Current. Genetic diversity was highest in the center of the range and lowest in the warm‐edge population. The adaptive SNP candidates revealed similar genetic structure patterns, with a spread of adaptive alleles across most warm (northern) populations. The lowest, but most unique, adaptive genetic diversity values were found in both warm and cool population edges, suggesting local adaptation but low evolutionary potential. Critically, genomic vulnerability modeling identified high levels of vulnerability to future environmental conditions in Tasmanian populations. Populations of kelp at range edges are unlikely to adapt and keep pace with predicted climate change. Ensuring the persistence of these kelp forests, by boosting resilience to climate change, may require active management strategies with assisted adaptation in warm‐edge (northern) populations and assisted gene flow in cool‐edge (Tasmania) populations.

## Graphical abstract

The dominant Australian kelp, Ecklonia radiata, was genotyped from 10 locations spanning a 2000 km latitudinal gradient along the east coast of Australia using ddRAD sequencing. Strong genetic differentiation was found between mainland Australia and Tasmania, while adaptive SNP candidates identified local adaptation at the species' range edges with low evolutionary potential and high genomic vulnerability in Tasmanian populations. Conservation strategies such as assisted adaptation and gene flow may be required to boost the resilience of Australian kelp forests to future climate change.

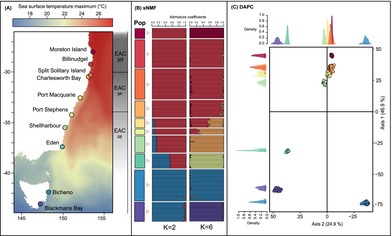

## 1 Introduction

Ongoing climate change significantly impacts the viability and persistence of wildlife populations (Hillebrand et al. 2018; Oliver et al. 2019; Ummenhofer and Meehl 2017). Global warming and increases in extreme weather events are pushing species beyond their physiological thermal thresholds, forcing them to move or go locally extinct (Rogan et al. 2023). As climate change reshapes natural habitats, conservation strategies that incorporate genetic information, such as assisted evolution, are crucial to enhance population resilience (Allendorf et al. 2010; van Oppen et al. 2015; Coleman et al. 2020). As species' ability to adapt to environmental change is influenced by evolutionary forces (Franks and Hoffmann 2012), adaptive genetic knowledge is essential to assess species' adaptive capacity to cope with climate change and forecast their persistence (Flanagan et al. 2018; Ofori et al. 2017) and represents a major tool for sustaining biodiversity under current and future climates.

Assessing species' adaptive capacity is vital for future‐proof conservation strategies (Coleman et al. 2020). This capacity depends on genetic variation present within and among populations (Franks and Hoffmann 2012), as well as non‐genetic factors such as ecological interactions, behaviour, and life‐history traits (Franks and Hoffmann 2012). Generally, high genetic diversity increases the likelihood to have individuals within a population carrying genetic variations encoding beneficial traits that can be selected for under changing environmental conditions (Hughes et al. 2008; Wernberg et al. 2018). Low genetic diversity on the other hand may indicate past selective pressure (Hampe and Petit 2005), maladaptation (Pearson et al. 2009), or genetic isolation (Diekmann and Serrão 2012). Genetic structure, shaped by factors like geographic and environmental isolation or species' life‐history traits (Balkenhol et al. 2019), can also influence how adaptive alleles can spread across a species' range (Lowe and Allendorf 2010). When planning conservation measures, integrating information on genetic structure further ensures long‐term success and allows tracking the distribution of adaptive variants (Balbar and Metaxas 2019; Wood et al. 2020). Such integrative estimation of genetic adaptive capacity is being increasingly applied to a wide variety of organisms (e.g., Jia et al. 2020; Silliman 2019; Van Daele et al. 2022; Vranken et al. 2021; Vu et al. 2020; Wood et al. 2021) but is yet to be commonly used at a management level (Taft et al. 2020). Given the alarming pace of climate change and associated population declines, it is critical to assess species adaptive capacity at large scales and inform managers on how this could be applied to assist natural systems in coping with future environmental conditions.

To assess the need for climate‐driven management interventions, genomic data can be used to predict the vulnerability of species to future climates, that is, the extent to which a species can genetically adapt at a sufficient pace to counter the projected rate of environmental change (Hoffmann and Sgrò 2011; Ofori et al. 2017; Sgrò et al. 2011). Among new predictive tools incorporating eco‐evolutionary processes (Thuiller et al. 2013), genomic vulnerability (cf. Bay et al. 2018), also referred to as genetic offset or genetic mismatch, measures the difference in genetic composition between the present genotype–environment association (GEA) and the genetic makeup required to sustain the population under predicted environmental conditions (Fitzpatrick and Keller 2015). It can help identify populations that are more vulnerable to climate change, where the rate of environmental change may outpace the ability to adapt through both mutation and selection over generations, necessitating prompt management interventions. Advances in sequencing and modelling (e.g., Gradient Forest, Generalised Dissimilarity Modelling) are making these predictions applicable at large spatial scales and on non‐model organisms (Balkenhol et al. 2019; Bierne et al. 2016). The estimation of genomic vulnerability to future environments is based on the extrapolation of local genetic information to any region for which we have environmental data (Capblancq et al. 2020; Derry et al. 2019). These features make genomic vulnerability a powerful tool for guiding conservation management strategies in a way that managers can visualise. However, interpretation for regions with limited genetic information should be approached with caution. This approach has been applied to predict the adaptive capacity of terrestrial species to climate change (e.g., Fitzpatrick and Keller 2015; Láruson et al. 2021; Ruegg et al. 2018; Sang et al. 2022) and is starting to be used on marine species (Adam et al. 2022; Vranken et al. 2021; Wood et al. 2021).

Genomic information on adaptive capacity and genomic vulnerability has important implications for conservation efforts aiming to harness natural genetic variability to support the persistence of a species under future climatic conditions (Razgour et al. 2018, 2019). This knowledge can guide the choice of appropriate management and climate interventions such as genetic rescue or assisted gene flow. Genetic rescue aims to increase the adaptive capacity of a population by introducing novel genotypes from other populations (increase in genetic diversity) which can be used to mitigate extinction in small, isolated, or maladapted populations (Bell et al. 2019). Assisted gene flow is the strategic transfer of genotypes from populations with high adaptive capacity to particular stressors to those with high levels of genomic vulnerability to the same stressor to boost future resilience to environmental change (Capblancq et al. 2020). While there are several examples where genetic information has informed proactive conservation or management strategies in terrestrial systems (Bertola et al. 2023; Forester and Lama 2022; Frankham 2015; Tokarska et al. 2011), crop management and farming optimization (e.g., Nicolia et al. 2014; Zhu et al. 2020), such approaches have not yet been applied in the context of in situ conservation of marine biodiversity (Coleman et al. 2020; van Oppen et al. 2015) but tools to achieve this are emerging (Wood et al. in press).

Marine ecosystems are increasingly being affected by climate change, with high vulnerability to warming in coastal ecosystems (Wernberg et al. 2024). In particular, kelp forests have undergone global declines (Krumhansl et al. 2016; Smale 2019; Wernberg, Krumhansl, et al. 2019) threatening the persistence of the ecosystem services and the unique biodiversity they support (Bennett et al. 2015; Vásquez et al. 2014). This decline is mainly caused by ocean warming, which occurs at a rapid pace, making implementing proactive conservation measures informed by genetics increasingly important for adaptation to future climates (Coleman et al. 2020; van Oppen et al. 2015). On the east coast of Australia, a global warming hotspot that is warming four times the global average (Hobday and Pecl 2014), warming has led to shifts in seaweed species distributions (Wernberg et al. 2011) and declines of kelp forests in Tasmania (Johnson et al. 2011) and New South Wales (Vergés et al. 2016). Given ongoing warming rates and the predicted extent of future kelp decline (Martínez et al. 2018, Davis et al. 2021), understanding the genetic basis of adaptive capacity and genetic structure in kelp populations can play a crucial role in developing proactive conservation strategies that will help kelps persist in the face of environmental change. Genomic vulnerability assessments of West Australian kelp forests have identified populations that lack adaptive capacity or show evidence of putative local adaptation (Vranken et al. 2021). However, such knowledge is lacking for *Ecklonia radiata* (C. Agardh.) J. Agardh, a major marine habitat‐forming species along the temperate east coast of Australia, a global warming hotspot.

In this study, we assess the genomic vulnerability of *E. radiata* kelp forests to climate change along the east coast of Australia using reduced representation sequencing. We aim to identify signatures of selection in genomic regions and their association with local environmental conditions and investigate how these vary across populations. We also aim to assess the role of genetic diversity and structure in shaping the adaptive capacity of these kelp forests. We model the genomic vulnerability of kelp populations to future climates to inform the development of novel conservation and management strategies aimed at boosting the resilience of kelps in changing climatic conditions.

## 2 Materials and Methods

### 2.1 Sample Collection

**FIGURE 1:**
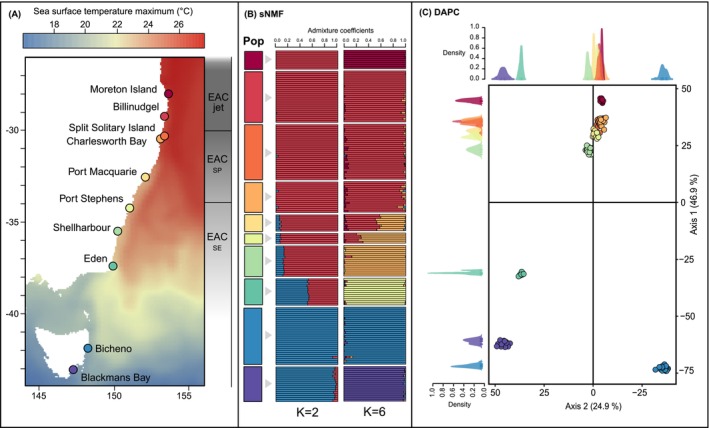
(A) Map of sampling locations on the east coast of Australia with sea surface temperature maximum and East Australian current (EAC) components (EAC jet, EAC separation point (EAC SP) and EAC southern extension (EAC SE)); (B) genetic structure LEA sNMF plot and map of the 10 sampled populations for *K* = 2 and *K* = 6 (optimal *K* for the neutral dataset); and (C) DAPC based on neutral loci using all populations. Density plots for the first and second discriminant axes as shown. Populations in the DAPCs are coloured by sites as on the map in (A).

251 *Ecklonia radiata* sporophytes were collected in 10 sites distributed along the eastern coast of Australia between January 2017 and September 2018. Sampling occurred across the entire species distribution range on the eastern coastline of Australia, from its warm edge in Queensland to its cold edge in Tasmania (~1900 km of coastline). This covered 16° of latitudinal gradient corresponding to an environmental temperature gradient of approximately 9.5°C based on mean annual sea surface temperature (Figure 1, Table S5). In each site, 17 to 30 kelp individuals separated by at least 1 m were haphazardly sampled within ~30 m^2^ out of consistency with previous work (Coleman et al. 2020; Vranken et al. 2021). Samples were collected between 2 and 15 m of depth via snorkelling or SCUBA diving depending on the kelp forest location and ease of access. Only kelps from Moreton Island (Queensland) were collected at 30 m due to the absence of kelp forests on shallow reefs (Davis et al. 2022; Marzinelli et al. 2015). Clean and healthy‐looking lateral tissue samples were snap frozen and stored at −80°C until DNA extraction.

### 2.2 DNA Extraction, Library Prep and ddRAD Sequencing

Kelp genomic DNA was isolated using the Qiagen DNeasy plant DNA and purified with the DNeasy PowerClean Pro Clean Up Kit using 50 to 100 mg of ground frozen material. The extracted genomic DNA concentration was measured using a Qubit fluorometer (Invitrogen), the quality was assessed by looking at the fragment size distribution on a Labchip GX Touch 24 (PerkinElmer) and quantified on a Nanodrop spectrophotometer (Nanodrop Technologies). One hundred sixty‐three individuals with high DNA quality were selected to proceed with the library process. We prepared four double‐digest restriction site‐associated DNA (ddRAD) libraries according to the modified protocol of Severn‐Ellis et al. (2020) as in Vranken et al. (2021). Each library had two to three technical replicates used to estimate sequencing and genotyping errors, with individuals from different sites scattered across libraries to minimize bias from library preparation. Paired‐end sequencing data was generated on Illumina high‐throughput sequencing platforms (on lane on the Illumina HiSeq X10 at the Garvin institute, NSW, Australia, two lanes on the Illumina HiSeq PE150 at the Novogene facility, Hong Kong, China, one lane on the NovaSeq X 10B lanes by AGRF, Melbourne, Australia, following the decommissioning of the HiSeq at Garvan and Novogene's transition to NovaSeq).

### 2.3 RAD Loci Assembly and SNP Calling

Following sequencing, ddRAD‐seq paired‐end reads were processed using the following bioinformatic pipeline to generate high‐quality SNPs. The quality of the raw Illumina reads was checked using FastQC version 0.1.11 (Andrews 2010) and multiQC version 1.0 (Ewels et al. 2016). Illumina raw reads BCL files were converted to FASTQ files using bcl2fastq version 2.9. Paired reads were demultiplexed using the STACKS version 2.5 “process_radtags” component (Catchen et al. 2013). Adapters were removed and reads were trimmed to 140 bp. Similar reads were assembled into loci using the STACKS “ustacks” component of the “denovo_map” pipeline. Based on previous work with a similar dataset (Silva et al. 2021; Vranken et al. 2021) and exploration of the impact of different combinations of key STACKS parameters ‘‐m’ and ‘‐M’ (Paris et al. 2017) using RADStacksHelpR on m = M with values ranging from 2 to 8 (DeRaad 2021), we concluded that m = M = 3 was producing the highest number of high‐quality SNPs and was used to perform variant calling and generate a catalogue of RAD tags (data not shown). The parameter ‘‐*n*’ was set to *n* = M, allowing for three mismatches between orthologous loci of different individuals during catalogue construction as recommended in Paris et al. (2017).

### 2.4 SNP Filtering and Impacts of Filtering

Following loci assembly and SNP calling on 171 individuals, which includes technical replicates, a series of filters was applied on the dataset using the STACKS “populations” module and VCFtools version 0.1.15 (Danecek et al. 2011). Those steps enable the retention of only high‐quality SNPs and limit the inferences of non‐biological signals that may arise from library construction, sequencing errors, depth of sequencing, or bioinformatic pipelines (Cerca et al. 2021; Mastretta‐Yanes et al. 2015; Shafer et al. 2017). The dataset used for downstream applications was constituted of only bi‐allelic sites, without indels, and with only one SNP per RAD locus to minimise strong linkage. The maximum missingness per site was limited to 10%, with a depth of coverage ranging between 5 and 30 to limit the SNPs derived from paralogous loci (Verdu et al. 2016). A minimum allele frequency of 0.05 was used (MAF > 5%), with prior look at the impact of different MAF on the number of private alleles (Table S2). Only SNPs present in 80% of sites and in at least 80% of individuals within those sites were kept, as advised by Paris et al. 2017. All SNPs not in Hardy–Weinberg equilibrium within more than 25% of the sampling sites (*p* < 0.001) were removed (dDocent, Puritz et al. 2014). Choosing the optimal filtering cut‐offs was assisted by calculating the mean depth of coverage per individual and per locus across all individuals, the proportion of missing data per individual and per site, and the population‐level averaged number of private alleles and expected heterozygosity at every step of the filtering process (Tables S1 and S2). This was done to control for any sudden changes in their values which could artificially inflate or diminish some signals. The potential bias due to a library effect was assessed by calculating the ‘SNP error rate’ which is the amount of difference between sequencing replicates (Mastretta‐Yanes et al. 2015) and was < 1% in our study. The removal of duplicates from the final dataset arising from samples employed as technical replicates was done by excluding replicates with the lowest mean depth and the highest degree of missingness. Finally, individuals with a high rate of missing data (> 30%) were removed resulting in a final dataset of 10,700 SNPs and 162 individuals (see Table S1 for final mean depth and missingness).

### 2.5 Environmental Predictors

Environmental data used for the GEA outlier detection was obtained from Bio‐Oracle2 (Assis et al. 2018). Several steps were undertaken to minimize the correlation between variables. Initially, average, maximum, minimum, and range sea temperature and current intensity were extracted for surface and maximum depth from our 10 sites, in addition to light intensity, salinity, nitrate and phosphate, and chlorophyll concentration. Environmental predictors with high correlation factors (∣*r*∣ ≥ 0.6) and high Variance Inflation Factors (VIF > 10) generated during the RDA using the *vegan* R package (Dixon 2003) were removed to ensure the absence of significant multicollinearity. The significance of the variables used for the RDA model was also calculated using an analysis of variance (ANOVA) permutation test using the vegan function anova.cca, which was run with 999 permutations. In the end, a set of three uncorrelated significant variables was selected: sea surface temperature maximum, sea surface temperature range, and maximum current intensity at the maximum bottom depth (Figure S1).

### 2.6 Outlier Detection Methods

Outlier detection was performed to separate candidate neutral loci from a putative adaptive set of loci under selection. To improve the robustness of outlier detection and limit false positive candidates, a total of four outlier detection methods were employed, each with distinct assumptions. This approach included two genetic differentiation methods (PCAdapt Luu et al. 2017; and Bayescan Foll and Gaggiotti 2008) and two GEA methods (RDA Oksanen et al. 2012; and LFMM Frichot and François 2015). The PCAdapt method relies on the use of principal component analysis (PCA) to assess population genetic structure and identifies outliers when a SNP is strongly associated with the first selected K principal components (Luu et al. 2017). The first six principal components were used, and a list of outlier SNPs was produced by using an empirical threshold (top 1%, see Duforet‐Frebourg et al. 2016) using the R package *PCAdapt*. BayeScan 2.1 detects loci under selection by decomposing *F*_ST_ values into a locus‐specific component, reflecting directional selection, and a population‐specific component, reflecting demographic balancing selection processes (Foll and Gaggiotti 2008). BayeScan was run using 20 pilot runs with 5000 iterations each followed by 5000 iterations with a burn‐in of 50,000, 10 thinning intervals, and 100 prior odds. Convergence of the chains was checked using the Heidelberg and Welch's convergence diagnostic. Outliers were identified in both components when the FDR was > 5%. The two GEA outlier detection methods used imputed data based on the ancestry values from the sNMF imputation method (Frichot and François 2015) and used sea surface temperature max and range as well as average current intensity at the bottom depth. The redundancy analysis (RDA) linear regression approach implemented in the R package *vegan* (Oksanen et al. 2012) was used. The significance of the RDA was calculated using an ANOVA permutation test in the function anova.cca of the R package vegan with 999 permutations. All significant RDA constrained axes were considered for the outlier detection (*p* < 0.05) and loci with a loading greater than ±2.5 SD were considered as candidates. *R* ^2^ was used to assess the portion of genomic variation explained by the environmental predictors. Partial RDA accounting for population structure and distance‐based RDA using Moran Eigenvector Maps (MEMs) based on the least cost path between sampled sites were tested, but results showed weak detection power, likely due to the strong correlation between population structure and environmental predictors in our dataset. LFMM, implemented in the R package *LEA* (Frichot and François 2015), was used to detect outlier candidates associated with single environmental predictors. The number of latent factors, allowing for the population structure, was set in this study to *K* = 6. The distribution of the *p*‐values using the lasso penalty method was visually inspected to meet statistical assumptions and genomic inflation factors (GIF). GIF used for the three predictors were 0.98, 0.73, and 1.0. An FDR of 0.001 was applied to limit false positives. The adaptive dataset was constituted of all outlier candidates to allow detection of weak selection signals.

### 2.7 Genetic Diversity and Structure

Both neutral and adaptive SNP datasets were used to assess patterns of genetic diversity across the entire coastline. Genetic diversity for each site was estimated using the number of private alleles (Np), the percentage of polymorphic loci (PL) and the mean observed heterozygosity (*H*_o_), expected heterozygosity (*H*_e_) and nucleotide diversity (π) generated by STACKS based on all loci (Catchen et al. 2013). Allelic richness (Ar) rarefied by the number of individuals genotyped was computed using the *allelic richness* function of the *hierfstat* R package. Significant levels of differences in expected heterozygosity among sites were assessed using the Monte Carlo exact test from the *hs.test* function of the *Adegenet* R package and 999 permutations (Jombart 2008), and a significance threshold of 0.005 after Bonferroni correction for multiple comparisons. For the neutral dataset specifically, inbreeding coefficients (*F*_IS_) (Weir and Cockerham 1984) were calculated for every site using the R package hierfstat and the *boot.ppfis* function to generate a 95% interval using 1000 bootstraps (Goudet 2005).

Genetic structure was assessed for the neutral and adaptive datasets using pairwise *F*_ST_, DAPC, sNMF STRUCTURE‐like analysis, and AMOVAs. Pairwise *F*_ST_ values (Weir and Cockerham 1984) were calculated using the ‘populations’ module of STACKS (Catchen et al. 2013). Discriminant analysis of principal components (DAPC) was performed in the *adegenet* R package (Jombart 2008). A sparse non‐negative matrix factorization (sNMF) analysis was used to visualise membership to different genetic clusters implemented in the *LEA* R package. The sNMF function was run with default parameters and 1000 iterations. Admixture proportions were visualised in the *pophelper* R package using the sNMF run with the lowest cross‐entropy (Francis 2017). Analysis of molecular variance (AMOVA) was performed on the neutral and adaptive datasets to compare levels of genetic diversity between individuals within a site and between sites. Additional AMOVAs were performed to assess the level of differentiation between the mainland and the Tasmanian populations, as well as between each pairwise comparison of populations. The AMOVA was performed using the *poppr* R package (Kamvar et al. 2014). The significance of covariance components and fixation indices was tested with 10,000 permutations. To find the optimum K (number of genetic clusters best representing the genetic variation present in the dataset), the Bayesian information criterion (BIC) generated using the function *find.clusters* from the *adegenet* R package was used, as well as the cross‐entropy criterion present in the *LEA* R package (Frichot and François 2015) where the best *K* displays low cross‐validation error (Figure S2). The cross‐entropy was estimated on 10 replicate runs generated for *K* = 1–10 with 10 replicate runs (Figure S4).

A mantel test implemented in the *adegenet* R package was used to test for the isolation‐by‐distance (IBD) hypothesis and was implemented using coastal water distances obtained using the *viamaris* function of the *melfuR* R package (Brauer 2019) and *F*_ST_/(1‐*F*_ST_) values between paired sites. Gene flow directionality and magnitude were estimated using the divMigrate function of the diveRsity package with 999 bootstraps (Keenan et al. 2013; Sundqvist et al. 2016). Jost's D, Nei's G_ST_, and Nm methods were used and were convergent in their results. Only Jost's D distance results are showcased in this manuscript.

### 2.8 Genomic Turnover and Vulnerability

To quantify and map how spatial distance and environmental variables are associated with genetic variation, we used generalised dissimilarity modelling (GDM) (Fitzpatrick and Keller 2015) implemented in the *gdm* package (Fitzpatrick et al. 2022) using environmental predictors from averaged bottom ocean floor. We fitted the GDM model using genetic distance (*F*_ST_) from the adaptive dataset constituted of 633 SNPs. This was shown to provide minimal performance advantages as opposed to neutral or using all SNPs (Lind and Lotterhos 2024). Benthic environmental predictors (temperature mean at the mean bottom depth “BO2_tempmean_bdmean”, temperature range at the mean bottom depth “BO2_temprange_bdmean”, current intensity mean at the mean bottom depth “BO2_curvelmean_bdmean”) were used based on data from 2000 to 2014. Geographic distance, automatically calculated as a straight line between two GPS points, was also factored in. For each variable, the shape of the I‐spline functions was inspected to understand at which rate the allele frequencies change along the environmental gradient (Mokany et al. 2022). The maximum value for every I‐spline partial function informed the relative contribution of each environmental predictor to the model and enabled the ranking of environmental predictors based on their relevance in shaping levels of genetic differentiation (Fitzpatrick and Keller 2015). The GDM functions ‘*gdm.transform*’ and ‘*predict*’ using the first three axes derived from a PCA were used to create a raster of allelic turnover for the studied area. The GDM function ‘predict’ was then used to project the model to 2050 and 2100 environmental conditions extracted from the Bio‐Oracle database. The RCP8.5 was used as the most representative of current emissions trends for 2050 (Schwalm et al. 2020) and for 2100 for a more conservative estimation. The resulting metric measured genomic vulnerability (Bay et al. 2018) as the amount of genetic change required to match predicted environmental change similar to *F*_ST_ values.

### 2.9 Candidate SNP Gene Ontology

Assembled stacks sequences containing SNPs from the adaptive dataset were annotated based on a BLASTX search against the NCBI *Ectocarpus siliculosus* protein set, with an e‐value cut‐off of 1E‐5 (Cock et al. 2010; Cormier et al. 2017). The *E. siliculosus* annotations were used to assign gene ontology (GO) terms to each stacks locus through the DAVID database platform (Huang et al. 2007). GO enrichment analysis was also performed using DAVID with the default parameters.

In addition to the adaptive set of SNPs, gene ontology was investigated for loci with a strong proportion of alternative alleles in the northern warm range edge population (i.e., Moreton Island) under potential weaker selection. To do so, using the full SNP dataset, allele frequencies of the alternative allele (f(A)) were generated for each population. SNPs of interest were identified by screening for loci with a f(A) ≥ 0.5 in the targeted population (Moreton Island), while also having f(A) ≤ 0.15 in all other populations. As this complementary method is prone to false positives (allelic patterns caused by neutral mechanisms such as demographic bottleneck and spatial isolation), the detected SNPs were not added to the putative adaptive dataset and were solely used to explore biologically relevant gene ontologies in loci showing signs of fixation in the warm‐edge population not necessarily represented by other outlier detection methods.

## 3 Results

### 3.1 Genotyping, Outlier Detection and Generation of SNP Datasets for Analysis

171 samples were successfully sequenced, and assembly of similar reads using Stacks optimised for the highest number of loci generated 5,024,587 SNPs (Figure S1). Following quality filtering (Table S1), 10,700 SNPs were kept, reducing the number of individuals to 162 across 10 populations, with an average of 16 individuals per population (min = 5 individuals, max = 28 individuals, Table 2). From this dataset, 633 SNPs (6%) were identified as candidate outliers by at least one of the four methods used (Table 1). The remnant 10,061 SNPs that were not detected by any outlier detection methods formed the putative neutral dataset. The majority of outliers were detected by only one method (572 SNPs, 89.5% of the adaptive dataset), with 63 SNPs shared by two methods and only 4 SNPs identified across three methods. 540 SNPs were detected by GEA detection methods, and 147 SNPs were detected by genetic distance‐based methods (Table 1). Bayescan detected 45 significant positive outliers and 377 SNPs under balancing selection. With the two GEA methods combined, 294 SNPs were strongly associated with temperature range, 136 with temperature maximum, and 147 with current intensity (Table 1). The following analyses are based on a neutral dataset composed of 10,061 SNPs and a putative adaptive dataset composed of 633 SNPs. Additionally, 350 SNPs were identified as having a strong proportion of alternative alleles solely in the warm range edge population.

**TABLE 1:** Number of candidate SNP loci under putative selection identified by four outlier detection methods (two ‘Genotype–Environment Association’ and two ‘Genetic differentiation’ methods).

Category	Method	SNPs	Temp max	Temp range	Current
GEA	RDA	312	124	136	52
	LFMM	252	12	170	96
Genetic differentiation	PCAdapt	103			
	Bayescan	45			
Sum of adaptive outliers		633	136	294	147

### 3.2 Neutral Genetic Diversity and Structure

Levels of neutral genetic diversity were low overall (mean He = 0.122), with expected heterozygosity (He) values among sites ranging from 0.061 to 0.195 (Table 2). A peak in genetic diversity was observed in a central population (Eden He = 0.195, Table 2) with a minimum at the northern warm rear edge site (Moreton Island He = 0.061, Table 2) reflecting a higher proportion of fixed loci across individuals. Comparisons of expected heterozygosity between adjacent sites were significant in most cases (*p*‐values < 0.05, Table S4) except between the Billinudgel and Split Solitary Island. Values of *F*_IS_ were small and close to zero, with no obvious case of strong inbreeding or outbreeding (Table 2, Table S3). Private alleles (Np) were high in both the warm and cold edges, with 215 Np in Moreton Island, and 329 and 174 Np in Bicheno and Blackmans Bay (Table 2). Private alleles were much rarer in all other mainland sites (Np = 0–6, Table 2).

**TABLE 2:** Population genetics summary statistics for the kelp *Ecklonia radiata* along the eastern coastline of Australia (10 populations, 163 individuals) for the neutral and adaptive subsets. Populations are listed from north to south.

# Pop ID	Long	Lat	*N*	Neutral (10061 SNPs)							Adaptive (639 SNPs)					
				Np	%Poly	Ho	He	Ar	π	FIS	Np	%Poly	Ho	He	Ar	π
Moreton Island	153.463	−27.067	9	215	19.085	0.067	0.061	1.156	0.064	−0.029	22	17.476	0.056	0.051	1.137	0.054
Billinudgel	153.564	−28.516	25	1	47.154	0.122	0.117	1.306	0.120	−0.010	1	47.847	0.142	0.139	1.347	0.142
Split Solitary Island	153.180	−30.239	27	1	49.201	0.118	0.116	1.306	0.119	0.015	0	50.564	0.111	0.110	1.297	0.112
Charlesworth Bay	153.143	−30.268	15	1	44.446	0.117	0.114	1.306	0.118	0.016	0	44.426	0.115	0.110	1.297	0.114
Port Macquarie	152.845	−31.594	8	0	39.000	0.117	0.112	1.310	0.120	0.016	0	36.531	0.106	0.104	1.287	0.111
Port Stephens	152.142	−32.718	5	1	34.471	0.130	0.114	1.321	0.127	−0.031	0	38.694	0.145	0.130	1.360	0.145
ShellHarbour	150.881	−34.593	15	2	54.955	0.151	0.149	1.389	0.154	0.022	3	45.701	0.147	0.134	1.340	0.139
Eden	149.908	−37.072	13	6	60.991	0.198	0.195	1.489	0.204	0.023	31	48.700	0.139	0.138	1.359	0.143
Bicheno	148.312	−41.867	28	329	37.100	0.119	0.114	1.272	0.116	−0.010	54	13.622	0.047	0.036	1.083	0.036
Blackmans Bay	147.329	−43.009	17	174	45.035	0.133	0.126	1.321	0.130	−0.005	9	20.358	0.059	0.054	1.136	0.056

*Note:* For the neutral dataset only, FIS: inbreeding coefficient (associated 95% confidence interval in Table S3).

Abbreviations: π, nucleotide diversity; %Poly, percentage of polymorphism; Ar, allelic richness; He, expected heterozygosity; Ho, observed heterozygosity; Np, number of private alleles.

Overall genetic differentiation was moderate (global neutral *F*_ST_ = 0.264) but varied among locations, with relatively small pairwise *F*_ST_ (p*F*_ST_) values between pairs of sites north of Eden (p*F*_ST_ = 0.017–0.250, Table 3), and higher values between any pair that included Eden or the Tasmanian sites (p*F*_ST_ = 0.208–0.633, Table 3). The smallest p*F*_ST_ values (p*F*_ST_ < 0.02) were observed in some of the most northern sites, with genetic differentiation between sites separated by 5 km similar to sites separated by 200 km of coastline (Charlesworth Bay—Split Solitary Island p*F*_ST_ = Split Solitary Island—Billinudgel p*F*_ST_ = 0.015, Table 3).

**TABLE 3:** Pairwise *F*_ST_ (p*F*_ST_) values between populations of the *kelp Ecklonia radiata* along the eastern coastline of Australia. Neutral p*F*_ST_ values are represented below the diagonal, and adaptive p*F*_ST_ values above. All comparisons are significant (*p* < 0.05) based on pairwise AMOVA tests.

Neutral\Adaptive	1.	2.	3.	4.	5.	6.	7.	8.	9.	10.
1. Moreton Island		0.165	0.161	0.203	0.246	0.381	0.313	0.560	0.703	0.631
2. Billinudgel	0.118		0.068	0.053	0.085	0.170	0.196	0.419	0.520	0.449
3. Split Solitary Island	0.106	0.017		0.027	0.048	0.144	0.154	0.390	0.468	0.395
4. Charlesworth Bay	0.133	0.019	0.017		0.063	0.159	0.165	0.400	0.497	0.423
5. Port Macquarie	0.189	0.049	0.046	0.056		0.146	0.132	0.398	0.530	0.459
6. Port Stephens	0.250	0.073	0.070	0.086	0.068		0.126	0.323	0.516	0.419
7. Shell Harbour	0.189	0.102	0.101	0.102	0.065	0.045		0.295	0.497	0.415
8. Eden	0.382	0.298	0.299	0.296	0.260	0.229	0.208		0.504	0.397
9. Bicheno	0.633	0.546	0.548	0.545	0.534	0.523	0.494	0.301		0.307
10. Blackmans Bay	0.591	0.497	0.498	0.501	0.489	0.475	0.441	0.235	0.174	

The AMOVA on all sites revealed significant genetic differentiation among all populations (78.7%, Table 4 for *K* = 10), which is largely explained by differences between the mainland and the Tasmanian populations (74.2%, Table 4 for *K* = 2) observed while intra‐cluster differentiation remained low (13.6%, Table 4 for *K* = 2). AMOVAs made on each possible pair of populations and associated Monte Carlo tests revealed significant genetic differentiation between all populations. Additionally, there was significant IBD among all sites (IBD_all sites_: *R* ^2^ = 0.83, mantel test: *p* = 0.001, IBD_mainland sites_: *R* ^2^ = 0.7, mantel test: *p* = 0.001).

**TABLE 4:** Analysis of molecular variance (AMOVA) of *Ecklonia radiata* in the 10 populations along the eastern coastline of Australia, with no genetic clusters (*K* = 10) or with genetic clusters assigned to mainland populations and Tasmanian populations (*K* = 2), using the neutral and adaptive SNP datasets.

	Neutral					Adaptive				
	df	Mean sq	*σ*	%	*p*	df	Mean sq	*σ*	%	*p*
No genetic clusters (*K* = 10)										
Among populations	9	23,019.42	1434.35	78.72	0.001	9	1082.64	67.57	81.46	0.001
Within populations	152	364.90	20.28	20.28	—	152	15.38	15.38	18.53	—
Total	161	1631.30	1799.25	100.00	—	161	75.04	82.95	100.00	—
Mainland/Tasmania (*K* = 2)										
Between regions	1	154,962.55	2237.25	74.24	0.001	1	5514.16	72.84	59.47	0.023
Among populations within regions	8	6526.52	411.33	13.65	0.001	8	528.70	34.27	27.98	0.001
Within populations	152	365.90	364.90	12.11	0.001	152	15.38	15.38	12.56	0.001
Total	161	1631.30	3013.48	100.00	—	161	75.04	122.28	100.00	—

Abbreviations: *σ*, variance component; %, percent variance; df, degree of freedom; Mean sq, mean of squares; *p*, *p*‐value.

**FIGURE 2:**
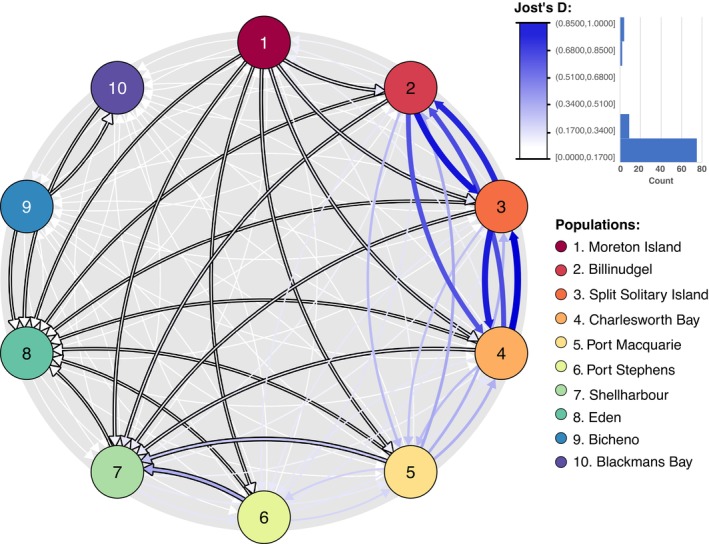
Migration rates (Jost' D metric) generated between each population. Migration rates are represented by arrows with values ranging from 0 to 1 and are colour coded accordingly with values closer to 1 being blue. Arrows with a black outline are showing significant asymmetric migration calculated from 999 bootstrap iterations. When migration rates are symmetrical between two populations, no arrow is outlined.

For the neutral dataset, the optimum number of genetic clusters was estimated to be *K* = 6 (Figure S2) which was retained for the sNMF STRUCTURE‐like approach (Figure 1) and can also be observed on the DAPC. Following a hierarchical approach from *K* = 2 to *K* = 10 (Figure S3), three main breaks can be identified. The first level of division, represented by *K* = 2 (Figure 1B) to *K* = 3 (Figure S3) showed a clear division between the mainland and Tasmania, with a high level of admixture in individuals from Eden, likely not reflecting true admixture, as Eden corresponds to a unique cluster with higher *K* values (Figure S3) and in the PCA. This is also found along the first axis of the DAPC representing 46.9% of the total genetic variation, where Eden and the Tasmanian populations are clustered together, with Eden still being clearly distinct from the Tasmanian populations (Figure 2C). The second DAPC axis, representing 24.9% of the total genetic variation, confirmed a second significant level of differentiation between the two Tasmanian populations, also shown with sNMF with no admixture (*K* = 4, Figure S3). The third significant genetic break separates the warm‐edge population of Moreton Island from the other mainland populations, as suggested by sNMF for *K* = 6 (Figure 2B) and the first axis of the DAPC (Figure 2C). The optimum number of genetic clusters, *K* = 6, further reveals some genetic admixture within the remaining mainland populations in Port Macquarie and Port Stephens, north of which a cluster is comprised of Billinudgel, Split Solitary Island, and Charlesworth Bay and south of which Shellharbour represents a distinct cluster, which can also be seen on the DAPC along the first axis.

Estimation of migration rates revealed extensive connectivity and bi‐directional dispersal north of the EAC separation point (located between 30° S and 34° S, Roughan and Middleton 2004; Ypma et al. 2016, Figure 1A). The highest migration rates were between Billinudgel, Charlesworth Bay, and Split Solitary Island, with Jost's D migration rates ranging between 0.68 and 0.1. Migration rates between the aforementioned sites and Port Macquarie ranged between 0.24 and 0.34 (Figure 2). South of the EAC separation point, migration rates are much lower, reduced to 0.18 and 0.17 between Port Macquarie and Port Stephens. Significant asymmetry in gene flow between paired populations (outlined in black arrows in Figure 2) almost consistently indicates a southward migration rate, apart from between the Tasmanian populations and Eden. Despite its weak values, Moreton Island had significant asymmetry in gene flow towards all mainland sites in a southward direction. Asymmetric gene flow also shows substantial values from Port Macquarie and Port Stephens towards Shellharbour, with migration rates of 0.22 and 0.32. Eden has a significant southward reach on all populations until Eden. Migration rates (±SE) between mainland populations and Tasmania are also extremely low (2.25E‐03 ± 7.17E‐04), in contrast to the higher values observed within mainland populations (1.74E‐01 ± 3.41E‐02), reflecting a 98.74% reduction in gene flow.

### 3.3 Putative Adaptive Population Diversity and Structure

When compared to the neutral dataset, the adaptive genetic diversity revealed similar overall low levels of diversity (mean He = 0.101), with similar results across all genetic diversity metrics, albeit with some differences in population rank (Table 2). The lowest adaptive diversity values were observed in the northern warm‐edge population (Moreton Island, He = 0.051, Table 2) and the two Tasmanian populations (Bicheno He = 0.036, Blackmans Bay He = 0.054, Table 2). This pattern was less pronounced for allelic richness, with relatively high allelic richness maintained in the warm‐edge population (Moreton Island Ar = 1.137, Table 2). The highest levels of adaptive genetic diversity were observed in Billinudgel (He = 0.139) and Eden (He = 0.138). The number of private adaptive alleles was highest at range edge populations: Moreton Island, Eden, and Bicheno (Table 2).

**FIGURE 3:**
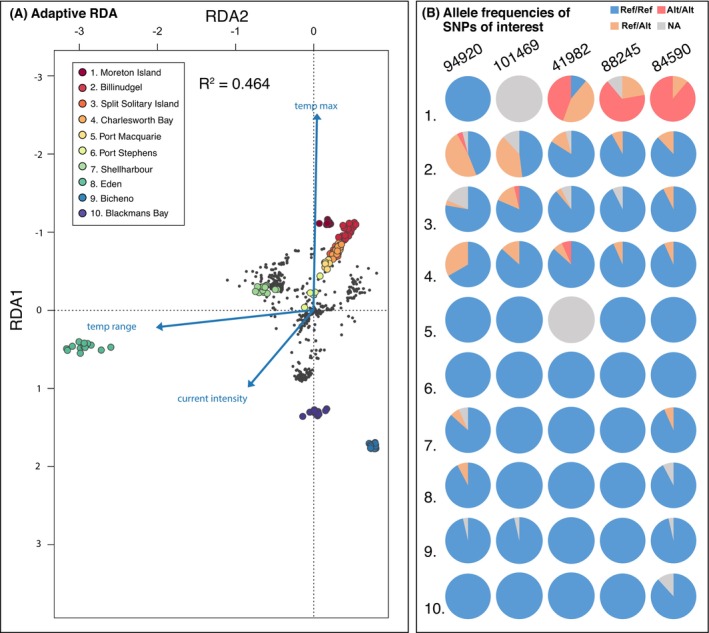
(A) Redundancy analysis (RDA) performed on the subset of 633 adaptive outliers on all individuals using three environmental explanatory variables (blue arrows) on the two first constrained ordination axes. Candidate SNPs are grey filled circles. Populations are coloured filled circles (B) Pie charts depicting allele frequencies of SNPs of interest. See Table 5 for corresponding information.

Overall genetic differentiation in the adaptive dataset was higher compared to the neutral dataset (global adaptive *F*_ST_ = 0.314) with significant *F*_ST_ values ranging from 0.027 to 0.703 between pairs of populations (Table 3). The overall increased *F*_ST_ translated to a higher variance among populations (81.5%, Table 3) compared to the neutral dataset and a strong IBD pattern (IBD adaptive: *R* ^2^ = 0.66, mantel test: *p* = 0.001). This increased level of genetic differentiation was also reflected in the optimal amount of genetic clusters which, for the adaptive dataset, varied between *K* = 8 and 10 (Figure S3). Nonetheless, direct comparisons of neutral and adaptive DAPCs showed overall similar spatial structure (Figure S4). The RDA based on candidate outliers explained 46% of the total variance, with clear population differentiation similar to the adaptive DAPC (Figure S4). Along the first axis, all mainland individuals except those from Eden showed a positive association with maximum temperature, while Eden and the Tasmanian populations showed a negative association with maximum temperature. The second axis primarily contributed to separating Eden from the remaining populations, with a strong association with temperature range (Figure 3). Current intensity had a lesser effect in clustering individuals compared to other environmental predictors but contributed to creating an axis along which individuals north of Shellharbour aligned (Figure 3).

### 3.4 Associated Function of Outlier Loci

Two datasets were compiled from the list of adaptive outliers and the list of SNPs with high fixation of the alternative alleles at Moreton Island. From the set of 633 assembled stacks loci containing adaptive SNPs, 29 could be annotated based on BLASTX homology to *Ectocarpus* proteins. The list of 350 Moreton Island SNPs had 13 gene candidates (full list in Table S7). A gene ontology analysis revealed that the majority of candidate loci were assigned to genes associated with ATP and membrane‐related functions with no clear relationship with stress‐responsive genes (Table S6). The GO terms related to stress response among candidate loci were limited to five genes associated with heat resistance, DNA repair, flagella, and other stress responses (Table 5), and associated allelic states are illustrated in Figure 3B.

**TABLE 5:** Gene functions associated with candidate SNPs based on the homology of stacks loci *to Ectocarpus* proteins (ADAPTIVE = locus identified as potential outlier; MORETON = locus with alternate alleles highly present in Moreton Island). See Figure 4B for associated allele frequency.

Subset	Locus	prot_ID	Evalue	Bitscore	Gene name	Relevance	INTERPRO top result
ADAPTIVE	CLocus_94920	CBN75052	1.93E‐08	50.1	C2H2 zinc finger protein	Plant C2H2 zinc finger proteins are mainly involved in plant growth and development and the responses to environmental stresses (Kim et al. 2015; Han and Fu 2019)	IPR013087: Zinc finger C2H2‐type/integrase DNA‐binding domain
	CLocus_101469	CBN75240	2.44E‐22	87.8	DEAD box helicase	Confer abiotic stress tolerance to plants by supporting mechanisms such as ROS scavenging, enhanced photosynthesis, ion homeostasis and regulation of various stress‐responsive genes (Nidumukkala et al. 2019)	IPR001650: Helicase, C‐terminal
MORETON	CLocus_41982	CBJ30979	1.83E‐26	99	Alb3 homologue, thylakoidal inner membrane insertase	Chloroplast insertase Alb3 orthologs are involved in light dependent interactions. Truncated Alb3 protein are known to generate mutants that can grow under low light conditions in *Arabidopsis thaliana* (Bellafiore et al. 2002; Urbischek et al. 2015; Qiu et al. 2018). Loss of this insertase also showed impacts in diatoms (Nymark et al. 2019)	IPR001708: Membrane insertase OXA1/ALB3/YidC
	CLocus_88245	CBJ27456	5.07E‐16	68.2	Ras superfamily GTPase (RTW)	Ras GTPase superfamily is known to be involved in male fertility with Ras‐related proteins involved in sperm motility (Lo et al. 2012; Bae et al. 2022)	IPR001806: Small GTPase superfamily
	CLocus_84590	CBN77905	7.94E‐13	62.4	SAM domain‐containing protein	Sterile alpha motif (SAM) is a large protein interaction domain involved in developmental regulation (Schultz et al. 2008), including in *Ectocarpus* (Pearson et al. 2019) with examples of genes involved in pheromone‐responsive mating (Klosterman et al. 2008)	IPR001660: Sterile alpha motif domain

### 3.5 GDM Modelling and Genomic Vulnerability

**FIGURE 4:**
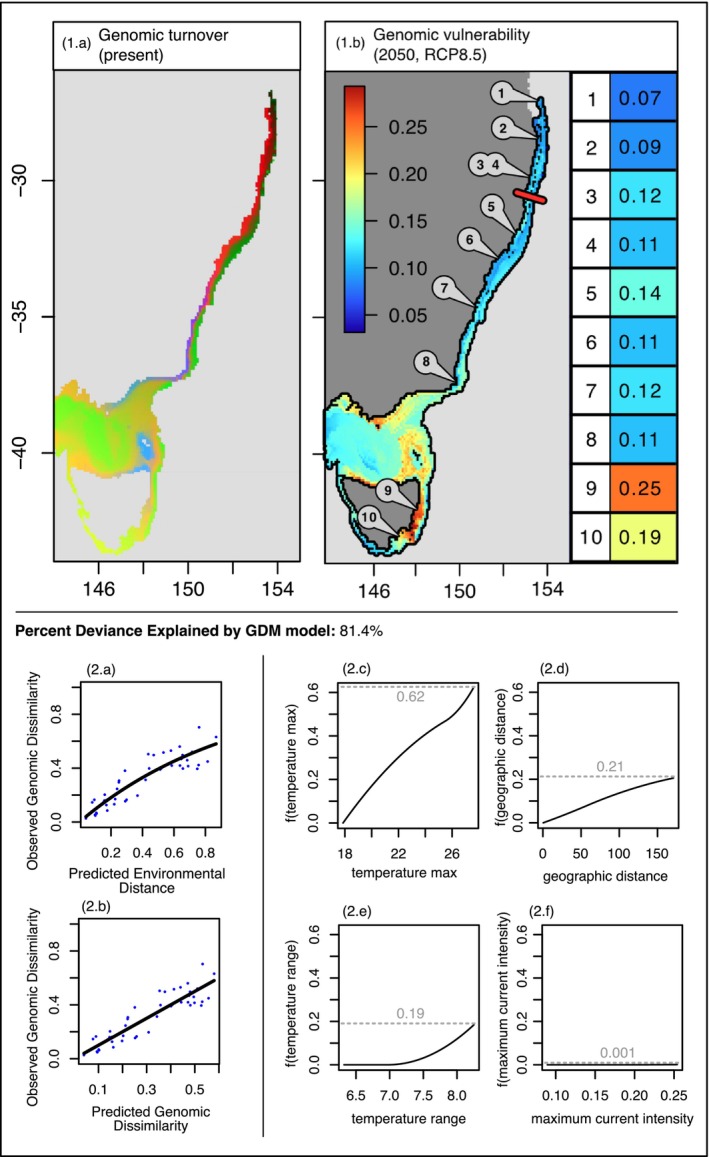
GDM model outputs and functions based on the adaptive dataset (1.a) GDM‐based predicted genetic turnover across *Ecklonia radiata*'s distribution along the east coast of Australia derived from transformed environmental predictor. Regions with similar colours are expected to harbour similar genetic composition. (1.b) Predicted genomic vulnerability for the year 2050 under the RCP8.5 scenario. on each map using a *F*_ST_ like unit. High values in red indicate high genomic vulnerability (turnover function of GDM indicates rapid changes in allele frequencies and indicates that more genomic change will be needed to adjust to future climate conditions), while blue values indicate little expected change. The black outline shows the species range of *Ecklonia radiata*. Site locations (1–10) are shown. The red bar indicates the projected northern range edge of the species by 2050 based on the averaged maximum temperature in Moreton Island from 2000 to 2014 (Bio‐ORACLE). Genomic vulnerability values were extracted for each site and are shown with a colour scale pairing the one used in the map. Panel 2 shows the GDM model (2.a,b) and spline functions for each environmental predictor (2.c–f). On the left section are found the relationship between predicted environmental distance and observed genomic dissimilarity obtained from site paired data (2.a), and the relationship between the predicted and observed genomic dissimilarity (2.b). On the right section (2.c–f) are shown the I‐spline functions used to fit the final GDM model for the four environmental predictors: Mean bottom depth temperature max, temperature range, maximum current intensity, and geographic distance. For each GDM spline function, the maximum height of the spline function is shown with a grey dashed line and indicates the importance of the predictor in explaining dissimilarities.

The GDM models successfully explained 81.4% of the overall genetic differentiation based on adaptive *F*_ST_ values (Figure 4). Temperature maximum was the main predictor in explaining the observed genomic dissimilarities (maximum height of the spline function equal to 0.62, Figure 4, panel 2.c). Geographic distance and temperature range were predicted to both have almost equal prediction power around 0.2 (Figure 4, panel 2.d and 2.e), while current intensity was close to being non‐significant (Figure 4, panel 2.f). The shape of the I‐spline function for the surface temperature maximum indicated a quasi‐linear function, with constant change in genetic differentiation as temperature maximum increased (Figure 4, 2.c). Beyond 26°C, the relationship became steeper, indicating that genetic dissimilarity increased more rapidly per unit change past this temperature (Figure 4, 2.c). Geographic distance also had a continuous linear relationship in explaining change in genetic composition. The allelic composition only started to change under the influence of temperature range past 7°C (Figure 4, 2.e).

The spatial projection of adaptive genetic variation showed a continuous genetic composition along the latitudinal gradient, with a coastal homogeneous cluster running from the northern edge of the distribution to Shellharbour (red cluster, Figure 4, 1.a), followed by a different cluster from approximately Shellharbour to Eden (purple cluster, Figure 4, 1.a), and a third cluster present further south, west of Eden, in Victoria (blue cluster, Figure 4, 1.a). This model also clearly highlighted a separation between coastal and offshore reefs, with the genetic turnover map showing distinct coastal and offshore clusters, with more variation within the coastal groups (Figure 4, 1.a).

Genomic vulnerability estimations by 2050 using the RCP8.5 scenario showed spatial variation in the magnitude of vulnerability. From north to south, a clear cline can be observed with a minimum genomic vulnerability observed in the northern regions and an increase in vulnerability values southwards (Figure S6). Regions with the highest levels of genomic vulnerability were the northern and eastern coastlines of Tasmania, as well as part of Victoria (Figure 4, 1.b). This pattern was consistent with the 2100 RCP8.5 predictions, which showed an increase in vulnerability throughout *E. radiata* 's distribution (Figure S5). In particular, by 2100, high values of genomic vulnerability > 0.2, previously contained to latitudes greater than—41° S by 2050, then started at—34° S (Figure S6). High genomic vulnerability in both Tasmanian sites (respectively 0.25 and 0.19 for the 2050 predictions, Figure 4) reflects strong environmental change (average environmental change estimations (av. ∆ 2050) equal to 0.91 and 0.86 for Bicheno and Blackmans, Table S6).

## 4 Discussion

To assess the adaptive potential of kelp forests in a global warming hotspot, we used reduced representation sequencing on the dominant kelp *Ecklonia radiata* to characterise neutral and adaptive genetic variation, gene flow, and genomic vulnerability. We found strong GEAs along Australia's eastern coastline, primarily with temperature. Both warm and cool edge populations exhibited low adaptive potential, with low levels of genetic diversity and high isolation. The neutral dataset showed strong genetic separation between mainland Australia and Tasmania, increasing the vulnerability of cool‐adapted Tasmanian populations. Candidate loci from the putative adaptive dataset and outliers specific to the warm edge were linked to known stress‐responsive gene functions, and genomic vulnerability was highest in parts of Tasmania. These findings provide novel information on intraspecific genotype‐climate association in kelp forests and a solid foundation for further experimental research on adaptation and inform conservation efforts.

### 4.1 Neutral Standing Genetic Diversity, Structure and Connectivity

Genetic diversity is vital to allow populations to adapt to environmental change (Lemopoulos et al. 2019; Matuszewski et al. 2015; Thrasher et al. 2018; Väli et al. 2008). Our study showed overall low levels of genetic diversity (average neutral H_e_ = 0.1), similar to that of *Ecklonia radiata* along the west coast of Australia (Vranken et al. 2021). Previous estimations on the east coast from Coleman, Chambers, et al. (2011) were higher but used microsatellites, which are often selected to be highly variable loci and therefore tend to inflate genetic diversity estimations (Fischer et al. 2017). Peaks in genetic diversity observed in mid‐latitudinal sites of Shellharbour and Eden could reflect the accumulation of migrants sustained by the predominantly poleward flow of the East Australian Current (EAC), a pattern found in other coastal species exposed to a boundary current (Coleman, Chambers, et al. 2011; Talbot et al. 2016; Triest et al. 2021; Wood et al. 2021; Nguyen et al. 2022). However, the relative isolation of Eden and strong genetic differentiation suggests additional contributing factors, such as north‐flowing currents, local retention mechanisms facilitated by eddies, or a potential secondary contact or refugia zone. The lowest level of genetic diversity was observed in the warm range edge population, Moreton Island. The low level of genetic diversity at the rear edge of the species distribution was expected and is consistent with previous genetic studies on *Ecklonia radiata* (Coleman, Roughan, et al. 2011; Vranken et al. 2021; Wernberg et al. 2018) and other kelp species (Guzinski et al. 2020). The low genetic diversity likely reflects genetic erosion, low effective population size, and genetic isolation (Hampe and Petit 2005). Factors involved are a combination of limited arrival of novel genetic information restricted by the poleward directionality of the EAC, habitat fragmentation, and population loss (Vergés et al. 2016), where fewer and patchier forests are being found closer to the species thermal maximum. The high retention of private alleles in this warm‐edge marginal population also supports high genetic isolation from other populations. These results corroborate the high number of private haplotypes found in the same population by Nimbs, Wernberg, et al. (2023) using a mitochondrial marker. Altogether, these results suggest both contemporary and ancient genetic isolation of this deep marginal population off Moreton Island, and its persistence at the species' warm edge might indicate adaptation to warmer conditions (Sgrò et al. 2011; Wernberg et al. 2018). While our results are inferred from populations with varying sampling sizes (5 to 28 individuals per population), and the detection of private alleles may be influenced by sampling bias, the use of a large SNP dataset provides robust genetic insights despite small sampling sizes (Nazareno et al. 2017).

Physical oceanography plays a structuring role in organizing the genetic composition of kelp forests (Coleman, Roughan, et al. 2011; Reynes et al. 2021; Ribeiro et al. 2022). Our estimations of genetic differentiation among the eastern kelp populations showed a lower global *F*_ST_ than the west coast (east coast *F*_ST_ = 0.264, this study; west coast *F*_ST_ = 0.411, Vranken et al. 2021). This aligns with previous microsatellite work that suggested that the characteristics of oceanographic currents, including their strength, directionality, and stability, determine seascape genomic structure, with the EAC being stronger than the Leeuwin Current (Coleman, Roughan, et al. 2011). Genetic clustering approaches revealed significant genetic separation (74%) between mainland populations and Tasmania. This genetic break was previously documented in other species of macroalgae (Fraser et al. 2009; Mueller et al. 2018; Wood et al. 2021), as well as in other temperate species associated with kelp forests (Klanten et al. 2020; Morgan et al. 2018; Ramos et al. 2018; Sinclair et al. 2016). In contrast, this differentiation was not observed in species with high dispersal and mobility capacity (e.g., Ashe and Wilson 2020; Thomas et al. 2021; Woodings et al. 2018). Tasmania is separated from the mainland by 220 km of open water, forming the Bass Strait, with an average depth of 60 m. However, the presence of numerous islands creates the potential for stepping stone connectivity. Strong genetic differentiation was also observed between the two Tasmanian populations, at a level not observed in other pairwise comparisons of populations located at a similar geographic distance on the mainland. This high genetic differentiation may be caused by the temporally dynamic influence of the EAC and associated eddies, the high complexity of the Tasmanian coastline with its intricate bays and complex small‐scale hydrodynamic processes (Durrant et al. 2018), and the influence of the Zeehan current on the southern coastline of Tasmania (Fraser et al. 2009; Weber et al. 2017).

High gene flow was quantified among sites north of the EAC separation point, particularly among Billinudgel, Split Solitary Island, Charlesworth Bay, and to a lesser extent, Port Macquarie. This area is highly exposed to the strong poleward flow of the EAC, which likely explains similar gene flow values in sites 200 versus 5 km apart. Across mainland populations north of Eden, *F*_ST_ values were generally less than 0.2, which indicates relatively high connectivity (> 1 migrant exchanged per generation; Mills and Allendorf 1996). Interestingly, despite the prevalence of the EAC off Moreton Island, limited gene flow with southward populations illustrates the genetic isolation of this marginal and deeper warm population. *Ecklonia*'s distribution starts to get patchy around Billinudgel (Davis et al. 2022) and is restricted to deeper waters north of Tweed heads (40 km north of Billinudgel and 140 km south of Moreton Island, Davis et al. 2022), it is likely that oceanographic connectivity prevents efficient dispersal from deep to shallow reefs on marginal reefs (Giraldo Ospina et al. 2023). Dominant southward but reduced gene flow from Port Macquarie south matches the pattern of strong admixture in Port Macquarie and Port Stephens, a region located between 31° S and 33° S where the EAC jet separates into the Tasman Front and the EAC extension (Cetina‐Heredia et al. 2014), characterised by the presence of cyclonic and anticyclonic eddies (Bowen et al. 2005; Oke and Griffin 2011). Altogether, in accordance with previous study on *Ecklonia radiata*, the EAC seemed to strongly affect genetic differentiation off Australia's east coast (Coleman et al. 2013, 2017; Coleman, Roughan, et al. 2011).

### 4.2 Adaptive Genetic Diversity and Structure

Our study identified outlier loci putatively under selection, identifying temperature maximum as the most important structuring predictor, based on the RDA and GDM models. This is no surprise given the role of temperature in governing the distribution of marine habitat‐forming species, including kelps, for which temperature regulates growth, phenology, and numerous metabolic processes (Wernberg et al. 2024). In our study, the maximum temperature varied by approximately 10°C across the species range. The genomic adaptive RDA showed a positive association between mainland northern populations (Moreton Island, Billinudgel, Charlesworth Bay, Split Solitary Island, Port Macquarie, Port Stephens and Shellharbour) and temperature maximum, as opposed to individuals from Eden and Tasmania, which were affiliated with cooler temperatures. While the northern mainland populations are under the influence of the warm east Australian boundary current (Roughan and Middleton 2004; Ypma et al. 2016), Tasmania's east coast is known to experience a strong influence of subantarctic waters with cold sea surface temperature (Harris et al. 1987, 1988).

Using the putative adaptive dataset, genetic diversity varied substantially across the sampled area, particularly with low values in both warm and cold edges (Moreton Island and the two Tasmanian sites). This was primarily explained by the fixation of alleles for most individuals in these populations, likely caused by directional selection or bottleneck events (Angert et al. 2020). It is important to note that allelic richness was relatively well preserved across all populations apart from Bicheno in Tasmania. This level of diversity represents an important evolutionary resource to respond to environmental change (Hoban et al. 2021, 2022). Furthermore, both the Moreton Island and the Tasmanian populations had substantial adaptive private alleles, which can contribute to adaptive capacity. This genomic signal of selection across a latitudinal gradient was also suggested for *E. radiata* along the West coast of Australia, with the lowest levels of adaptive genetic diversity (heterozygosity) observed in the warm‐edge populations (Vranken et al. 2021). This signal of genetic selection correlated with previously documented metabolic differences in kelps along the same gradient, and differences in resilience to marine heatwaves (Staehr and Wernberg 2009; Wernberg et al. 2018).

Given that populations north of Port Stephens are all experiencing temperatures in summer beyond the species temperature optima of 24°C (Castro et al. 2020; Wernberg, Coleman, et al. 2019), directional selection across extended parts of the coastline is expected. Among our outlier candidates (Figure 3), some alleles were shared across Billinudgel, the Split Solitary Islands, and Charlesworth Bay (e.g., loci 94920, 101469). Furthermore, despite the genetic isolation observed in Moreton Island, loci that showed a high proportion of homozygotes for the alternative allele in Moreton Island also had alternative alleles spreading in southern populations down to Charlesworth Bay. This coincided with the observation of high genetic connectivity within the EAC jet until reaching the EAC separation point (Figure 1). The occurrence of high adaptive genetic diversity in Billinudgel suggested that high gene flow does not necessarily prevent the maintenance of genetic variation relevant to adaptation (Bontrager and Angert 2019; Kottler et al. 2021). However, the strong homogenizing role of the EAC likely reduced the retention of private alleles, as evidenced by their low presence along the main path of the current. Signs of local adaptation were not restricted to the warm edge. The high putatively adaptive genetic diversity found in the southern mainland populations of Shellharbour and Eden may reflect the high environmental fluctuations experienced in this region, which can equally shape genomic variation (Chen et al. 2022).

Among the outlier candidates, two loci (Locus 94920 and Locus 101469, Table 4) showed polymorphism primarily in Billinudgel, and to a lesser extent in Charlesworth Bay and the Split Solitary Island populations. Both loci were associated with genes regulating abiotic‐stress‐related responses, such as temperature change induced stress, respectively the C2H2 zinc finger protein and a DEAD box helicase (Han and Fu 2019; Kim et al. 2015; Nidumukkala et al. 2019). In addition to loci detected by classic outlier detection methods, the gene ontology of SNPs showing a high proportion of alternative alleles only in Moreton Island was also investigated to further explore the hypothesized adaptation on the warm edge. Findings associated with those loci need to be interpreted with caution given the high chance of false positives with allelic frequencies influenced by demographic factors, such as isolation or bottleneck, not just local adaptation. Among the loci that showed a high proportion of the alternative allele in Moreton Island, locus 41982 was associated with the gene Alb3 homologue, a chloroplast insertase for which mutants are known to maintain growth under low light conditions in *Arabidopsis thaliana* (Bellafiore et al. 2002; Qiu et al. 2018; Urbischek et al. 2015). Given the presence of kelp forests in Moreton Island at depths greater than 30 m, this could represent an important adaptive trait. Other gene functions found in the Moreton Island outliers were associated with microtubules (loci 130161, 134324 and 1523428, Table S7) and sperm motility (locus 88245, Lo et al. 2012; Table 5; Bae et al. 2022), which could be relevant in ensuring sperm vitality under warm temperatures. Given that functional annotation for many candidate loci was unclear (the majority linked to ATP‐binding and membrane‐related activities), this highlighted the limitation of current genomic resources in identifying gene functions. Only a small proportion of the outlier loci had successful BLAST hits with gene names (31/633 SNPs), with many annotations restricted to general functional domains. Developing an annotated reference genome for *Ecklonia radiata* or using genomes from more closely related species of brown algae (Phaeoexplorer; Brillet‐Guéguen et al. 2023), combined with experiments to identify causal links, would help identify genes underlying adaptation.

Our putative adaptive dataset shared numerous features with the neutral dataset, especially genetic structure suggesting that both adaptive and neutral mechanisms occur along the same gradient. Ultimately, fixation of alleles could equally be caused by selection or neutral processes such as allele surfing, stochastic variation, migration, demographic changes, or strong isolation‐by‐distance (Alleaume‐benharira et al. 2006; Liggins et al. 2019; Lotterhos 2023). This can result in a change in allele frequencies obscuring the detection of outliers and leading to detecting false positives (Bierne et al. 2011; Rellstab et al. 2015). The GDM model revealed geographic distance as the second most important predictor, which illustrated how spatial and environmental gradients can play similar roles in structuring genetic composition across large‐scale latitudinal gradients. While paired sampling designs could help limit problems associated with spatial correlations for some species or geographic settings (Lotterhos and Whitlock 2015), this approach proved challenging along the eastern coastline of Australia due to its narrow latitudinal coastline and shelf. This prevented the identification of unconfounding environmental gradients that do not correlate with latitude (Nimbs, Champion, et al. 2023). Future research on *E. radiata* in Australia will apply the same ddRAD methods across both eastern and western coasts to provide new insights into adaptation across genetically distinct populations experiencing similar environmental gradients. Furthermore, while this study primarily focused on temperature, other environmental variables might be equally important for which data was not readily available, or which were correlated with temperature and therefore omitted (such as nitrate and phosphate concentrations).

### 4.3 Genomic Vulnerability

Estimations of genomic vulnerability differed greatly between the warm and cold edges, with low genomic vulnerability at the warm edge and a high genomic vulnerability around the northern and eastern coastlines of Tasmania. This reflects differences in the magnitude of projected environmental change (Table S5). While both regions are expected to experience substantial warming, the rate of change is relatively lower at the warm edge, where populations are already nearing their thermal thresholds, whereas southern populations are predicted to experience a broader amplitude of environmental change. Indeed, despite low genomic vulnerability for the northern mainland edge populations, eminent warming will likely exceed the species physiological upper thermal threshold by the end of the century (Davis et al. 2022; Martínez et al. 2018), rendering our genomic vulnerability analyses moot for this area. The southern populations from Tasmania, on the other hand, are at high risk of genomic vulnerability. This can seem counterintuitive as Tasmania is predicted to be one of the last places with suitable thermal conditions to support kelp populations (Martínez et al. 2018). Yet, our study shows that the anticipated environmental change will be higher in magnitude in Tasmania than in mainland coastal populations (Table S5), and that such drastic change in environmental conditions will likely require a substantial change in genetic composition that will be difficult for natural populations to achieve. Given the strong association of Tasmanian kelps with cold temperatures, their high genetic differentiation from mainland individuals, the restricted gene flow among Tasmanian populations, and the low adaptive genetic diversity (both expected heterozygosity and allelic richness), there are concerns about the long‐term survival of Tasmanian kelps from a genomic perspective, providing a different point of view than previous distribution model predictions (Martínez et al. 2018). Studies on kelp recruits and gametophytes show evidence of strong intraspecific phenotypic divergence in response to temperature (Britton et al. 2024; Veenhof et al. 2023; Alsuwaiyan et al. 2021). Recent work on *Ecklonia* recruits from Tasmania showed a thermal optimum considerably lower than the one previously obtained from warmer locations (Britton et al. 2024; Wernberg et al. 2016). Differences in intraspecific thermal tolerance in gametophytes from Coffs Harbour, Sydney, and Tasmania revealed that Tasmania harboured the lowest level of thermal tolerance (Veenhof et al. 2023). Therefore, further investigating intraspecific genetic differences within *Ecklonia* will likely not reduce the previously predicted decline in kelp forests (Castro et al. 2020; Davis et al. 2022; Martínez et al. 2018), but instead identify new regions where vulnerability will be driven by genetic differentiation. Genomic vulnerability modelling approaches, however, are far from perfect, and the incorporation of genetic connectivity in future models should refine vulnerability predictions. Furthermore, mesocosms and reciprocal transplant experiments are needed for further elucidating the causal relationship between climate‐vulnerable phenotypes and genotypes and exploring potential interventions to mitigate the anticipated loss of kelps in Tasmania.

### 4.4 Future Direction and Conservation

This work has important repercussions for designing management strategies that consider adaptive processes and how they can be harnessed to alleviate climate‐mediated declines. We identified genetic structure within *Ecklonia*'s range which can be used to design optimal sourcing for restoration purposes and genetic conservation in the event of local loss of kelps (Wood et al. 2024). The warm‐edge population of Moreton Island, where kelp only persist at depth > 30 m, showed low adaptive capacity, high isolation and genetic erosion, rendering it highly vulnerable to climate change. With temperatures set to exceed the species' physiological threshold, range contraction is imminent (Davis et al. 2022), highlighting the need for urgent conservation efforts. Considering the high genetic endemism potentially linked to higher thermal tolerance (this study and Nimbs, Wernberg, et al. 2023), preserving the adaptive potential of this population by setting up banks of gametophyte material should be prioritized. This will also facilitate experimental assessment of the thermal vulnerability of this remote marginal population, as well as be harnessed for future assisted adaptation strategies in vulnerable populations further south (Coleman et al. 2020). Similarly, the high putative adaptive diversity of populations from Billinudgel to Port Macquarie, strongly associated with high temperature, should equally be harnessed in biobanking and assisted adaptation strategies. Given the high genetic connectivity in most mainland populations, the spread of adaptive variants south of Port Macquarie should happen across large scales over generations without interventions as the EAC intensifies. Critically, the vulnerability of kelp in Tasmania requires the most attention. While it was understood that high connectivity present along the eastern coastline of Australia (Coleman et al. 2017; Coleman, Roughan, et al. 2011) could sustain a natural spread of more thermo‐tolerant kelps to higher latitudes, the presently described high genetic differentiation between mainland Australia and Tasmania and significant reduction in gene flow (98.7%) greatly limits this assumption. Instead, the introduction of genotypes from mainland populations into Tasmania could be envisioned to foster adaptive responses to predicted environmental change, which could combine genetic boosting and assisted gene flow approaches (Aitken and Whitlock 2013). Given the highly diverse nature of genotypes along the mainland, a composite sourcing approach could be undertaken to maximize climate matching (Jia et al. 2020). While genetic pollution and maladaptation are flagged as risks associated with assisted gene flow strategies (Ricciardi and Simberloff 2009), the limited connectivity found between the two Tasmanian sites indicates a low risk of the spread of introduced genotypes. Confirming limited dispersal along the coast of Tasmania requires further fine‐scale population genomics research. Experiments to validate reproductive success when crossing individuals from Tasmania with the selected donor sites, as well as assessing the fitness of the produced first generation, should first be assessed on gametophyte cultures to best identify seed sourcing strategies while addressing the risks of genetic pollution, maladaptation, and inbreeding or outbreeding.

## 5 Conclusions

Genomic information can help assess the adaptive potential and vulnerability of wild populations to climate change and can be incorporated into conservation plans that seek to future‐proof populations against climate change. This study revealed that kelp forests across the eastern coastline of Australia have low adaptive capacity at both their warm and cold edges of distribution, with regions in Victoria and Tasmania predicted to be under high genomic vulnerability to climate change. The role of the EAC in homogenising genetic composition, and the strong genetic break separating mainland populations from Tasmania, was further validated with important repercussions for sourcing individuals for restoration and planning assisted adaptation strategies. This study provides a novel basis for conservation‐informed decisions about kelps in a global warming hotspot and lays a foundation for further manipulative experiments linking genomic information with phenotypic responses to thermal stress.

## Author Contributions

**Antoine J. P. Minne:** conceptualization (equal), data curation (equal), formal analysis (lead), methodology (lead), resources (equal), visualization (lead), writing – original draft (lead), writing – review and editing (lead). **Sofie Vranken:** conceptualization (supporting), methodology (equal), resources (supporting), supervision (supporting), writing – review and editing (equal). **David Wheeler:** data curation (supporting), formal analysis (supporting), software (supporting), writing – review and editing (supporting). **Georgina Wood:** conceptualization (supporting), formal analysis (supporting), methodology (supporting), validation (equal), writing – review and editing (equal). **Jacqueline Batley:** methodology (supporting), resources (equal), supervision (supporting), writing – review and editing (supporting). **Thomas Wernberg:** conceptualization (supporting), funding acquisition (lead), supervision (lead), writing – review and editing (supporting). **Melinda A. Coleman:** conceptualization (equal), funding acquisition (lead), methodology (supporting), resources (equal), supervision (lead), writing – original draft (supporting), writing – review and editing (equal).

## Conflicts of Interest

The authors declare no conflicts of interest.

## Supporting information


Appendix S1.

## Data Availability

The datasets presented in this study can be found on Figshare: https://doi.org/10.6084/m9.figshare.28497356.v1. Any other relevant data supporting the findings of this study will be available from the corresponding author upon reasonable request.
