# Supplementary material for: Strong Environmental and Genome‐Wide Population Differentiation Underpins Adaptation and High Genomic Vulnerability in the Dominant Australian Kelp (Ecklonia radiata)
Source: Ecol Evol. 2025 May 12;15(5):e71158. doi: 10.1002/ece3.71158 (PMC12068950; doi:10.1002/ece3.71158)
Supplement: Supplementary file 1 — Appendix S1. [file ECE3-15-e71158-s001.docx]

## Supplementary materials


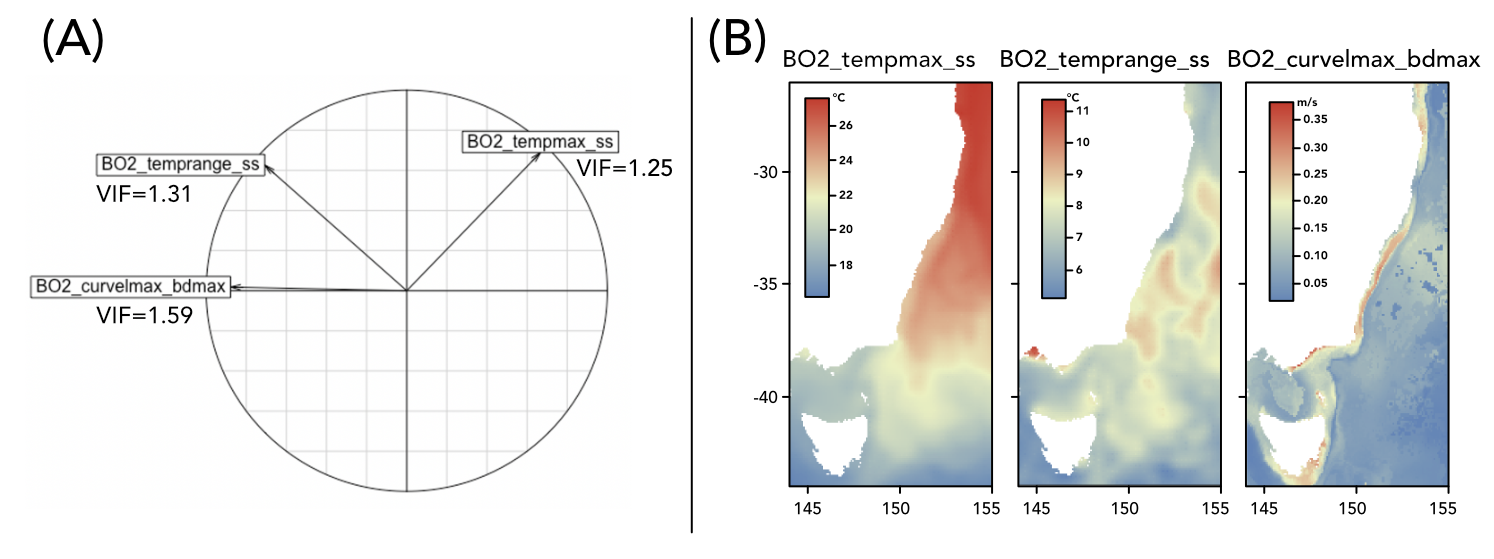


**Figure S1:** (A) Correlation circle with the final set of environmental predictors and associated variance inflation factor (VIF), (B) Spatial distribution of the selected environmental predictors across the eastern coastline of Australia relevant to *Ecklonia radiata* kelp forests. Data comes from BioOracle and was used for the RDA and LFMM outlier detection methods.


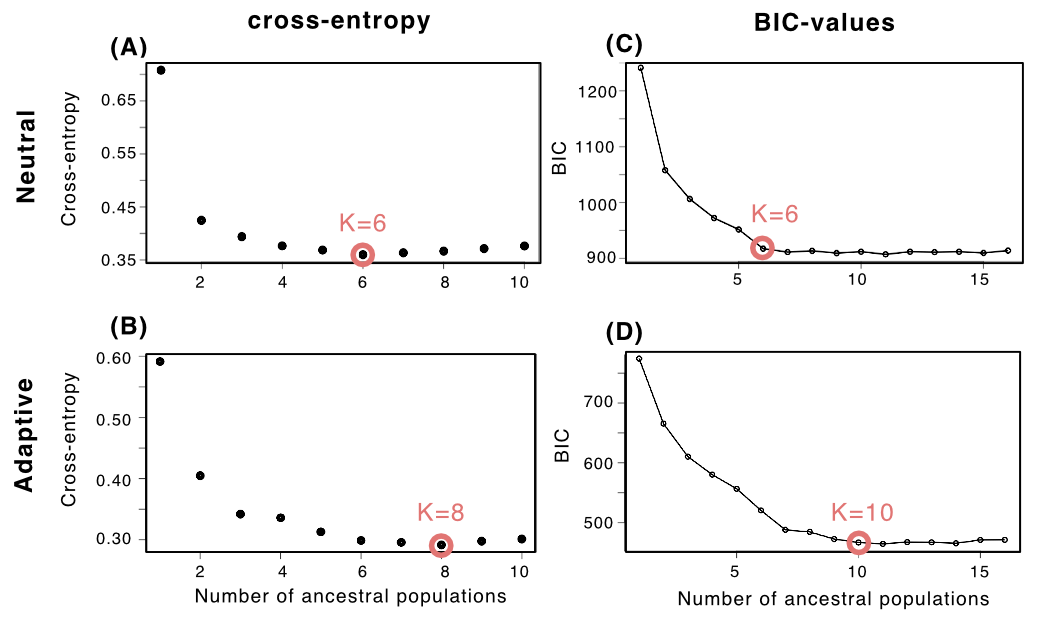


**Figure S2:** Estimations of genetic clusters for SNPs datasets using the cross-ancestry value method implemented in the sNMF function of the *LEA* R package (A-B) and the Bayesian Information Criterion (BIC) according to the number of retained clusters in the DAPC using the *find.clusters* function of the *Adegenet* R package (C-D). Estimations were made for both neutral (A-C) and adaptive (B-D) datasets. In the end, K=6 was kept for the neutral dataset and K=10 for the adaptive dataset. For A) and B) K associated with the lowest cross-entropy or BIC were considered as best K candidates.


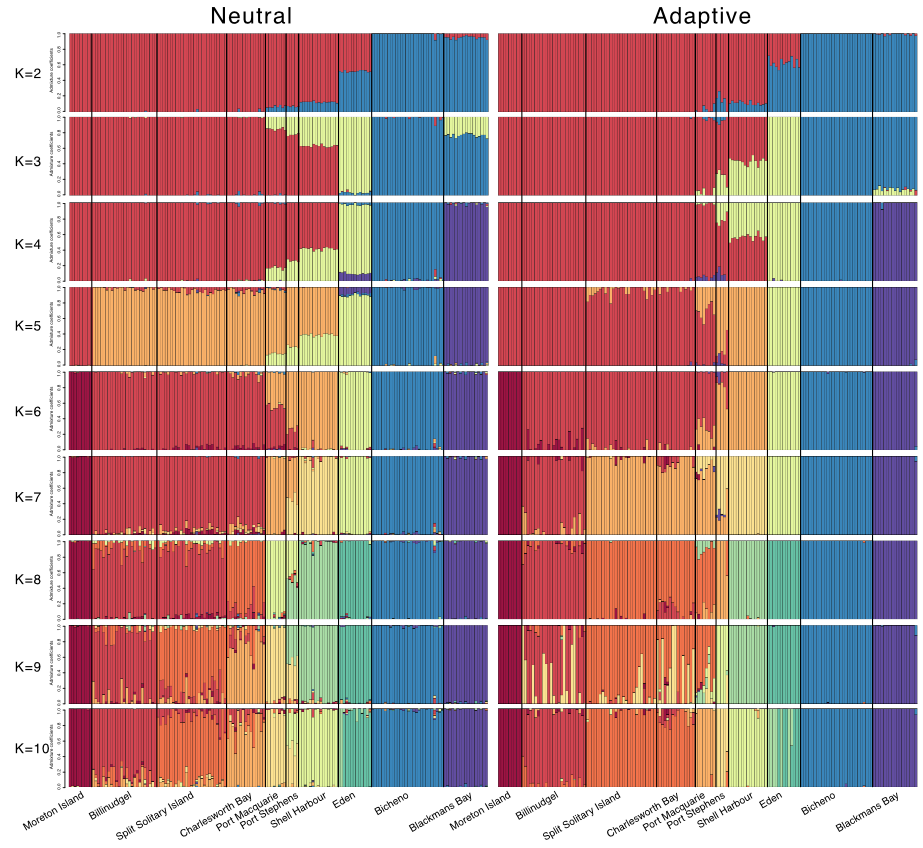


**Figure S3:** Hierarchical summary of all the sNMF runs from K=2-10 of 158 Ecklonia radiata samples from 10 sites using 8,426 neutral SNPs and 354 adaptive SNPs. Each individual is represented by a vertical line. The colour represents the membership probability to a K cluster and matches the colours used for sites in figure 2 and. X-labels indicate the individual labels.


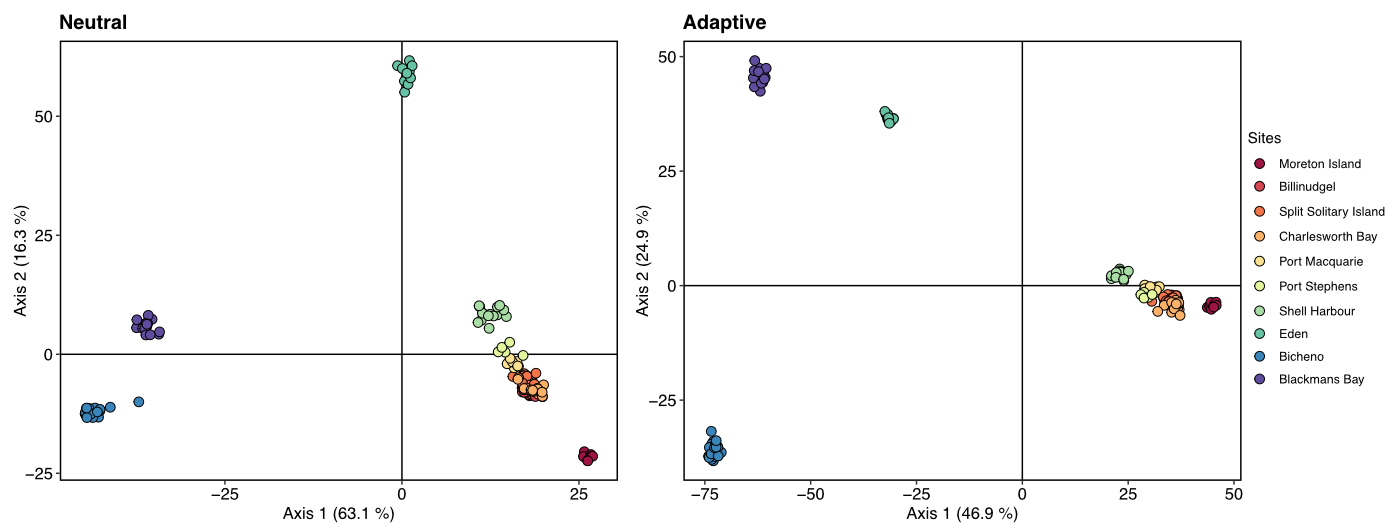


**Figure S4:** Discriminant analysis of principal components (DAPC) based on all populations using the neutral (left) and adaptive (right) SNP datasets. The 40 first PCs were used for the neutral DAPC and the first 30 PCs were used for the adaptive DAPC based on lowest Mean Squared Error.


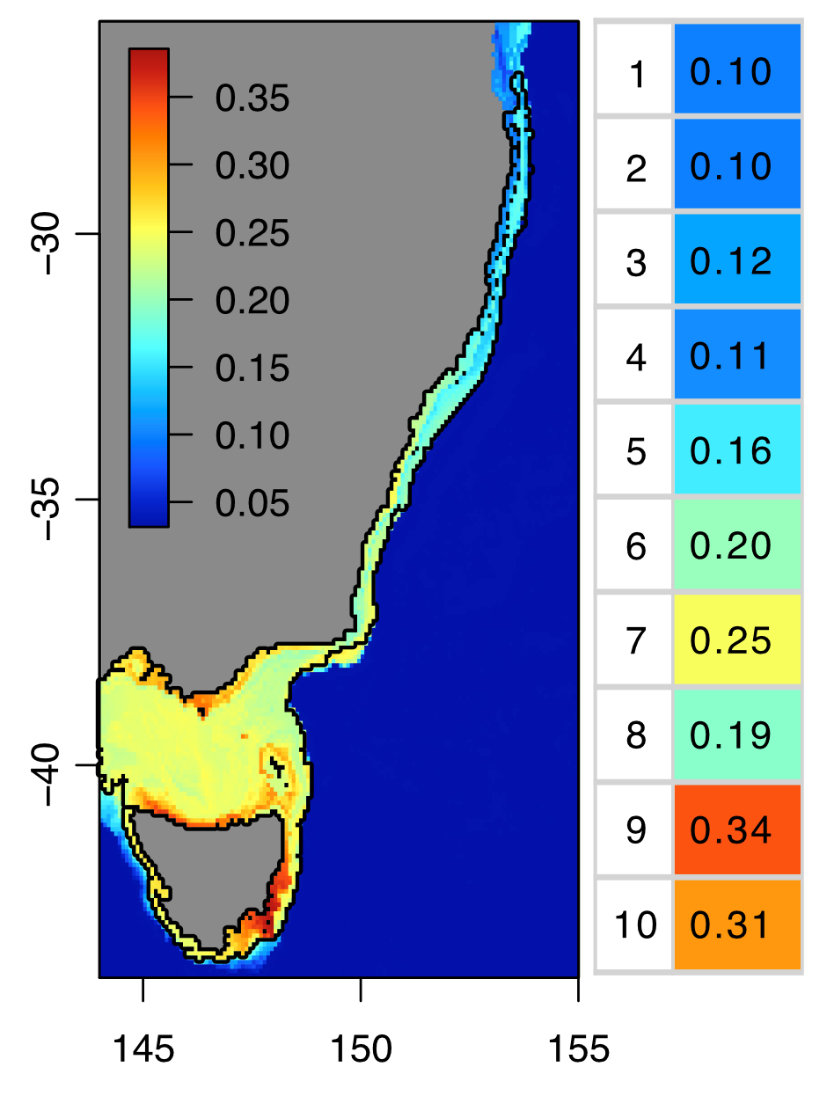


**Figure S5:** Predicted genomic vulnerability for the years 2100 under the RCP8.5 scenario. on each map using a F_ST_ like unit. High values in red indicate high genomic vulnerability, while blue values indicate little expected change. The black outline on every map shows the species range of *Ecklonia radiata*. Genomic vulnerability values for each site (1-10) were extracted.


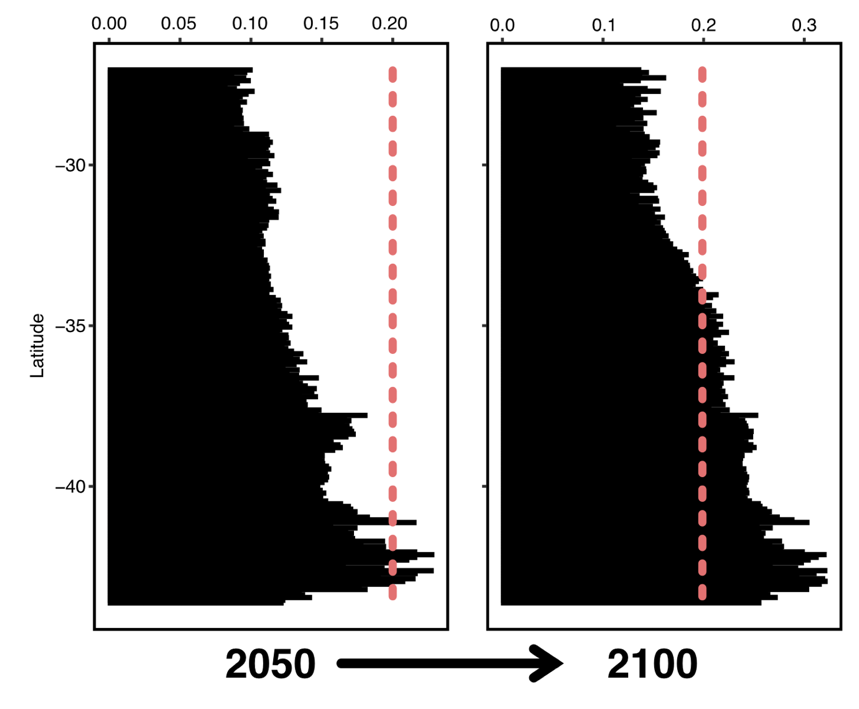


**Figure S6:** Averaged predicted genomic vulnerability (x-axis) for the years 2050 (left) and 2100 (right) under the RCP8.5 scenario across latitude (y-axis) based on ocean bottom floor (bottom) predictors. Values were extracted within the species distribution range of Ecklonia radiata along the east coast of Australia as in figure 4. The red dotted line represents a subjective threshold of genomic vulnerability set to 0.2.

**Table S1**: Quality filters applied to build the set of high quality 10,816 SNPs used. The filtering steps are followed from top row (n=5,024,587 SNPs) to bottom (n=10,700 SNPs). Number of SNP retained after each filtering step displayed. MAF from 2 to 10% are shown for comparison but MAF 5% was kept for downstream analysis. Missingness and depth values are means.


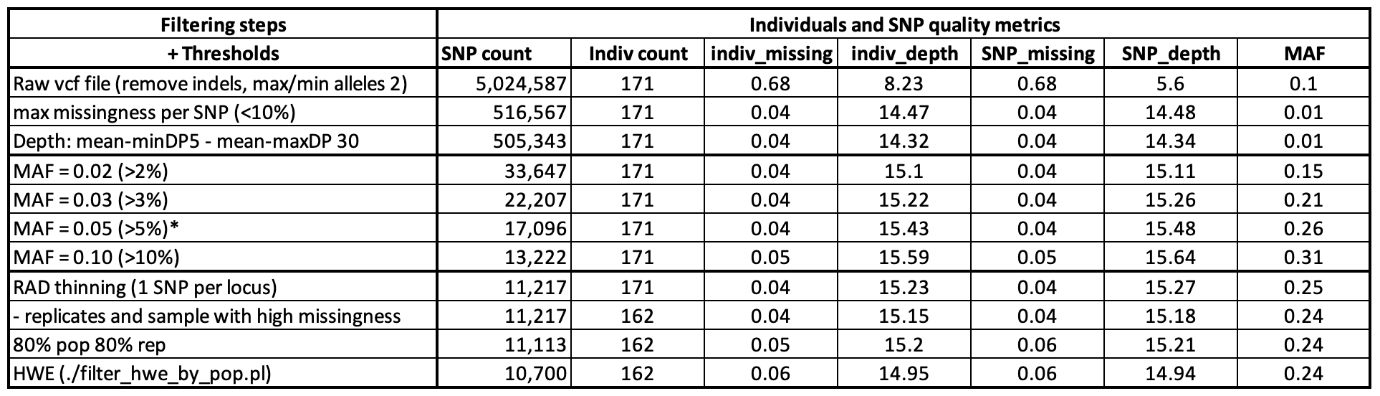


**Table S2**: continuity of Table S1 with information on the number of private alleles and expected heterozygosity across the 10 populations. The gradient colour codes show the highest values in green and the lowest in red for the Number of private alleles and Expected heterozygosity. MAF from 2 to 10% are shown for comparison but MAF 5% was kept for downstream analysis.

*
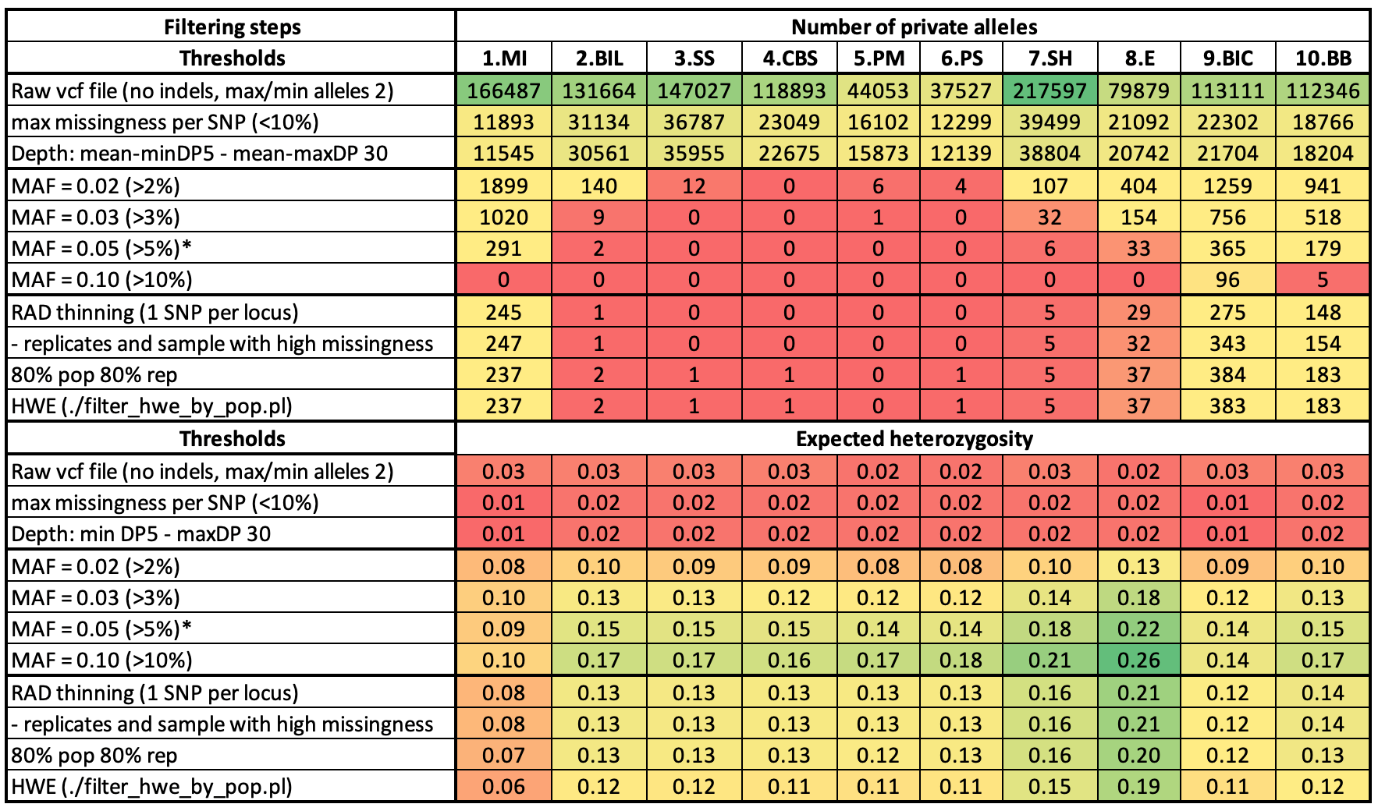
*

**Table S3**: FIS estimation with a 95% confidence interval on the neutral dataset.

| Population | FIS | [95% CI] |
| --- | --- | --- |
| Moreton Island | -0.029 | [-0.060,-0.019] |
| Billinudgel | -0.011 | [-0.031,-0.013] |
| Split Solitary Is. | 0.015 | [-0.003,0.015] |
| Charlesworth Bay | 0.016 | [0.001,0.022] |
| Port Macquarie | 0.016 | [0.013,0.042] |
| Port Stephens | -0.031 | [-0.039,-0.008] |
| Shellharbour | 0.022 | [0.013,0.033] |
| Eden | 0.023 | [0.017,0.036] |
| Bicheno | -0.010 | [-0.035,-0.013] |
| Blackmans Bay | -0.005 | [-0.030,-0.008] |

**Table S4:** Summary of the Monte-Carlo exact test with 999 simulations testing for differences (two-sided) in expected heterozygosity between pairs of two populations. Differences in expected heterozygosity and simulated p-values are presented. All populations were compared to their closest populations from north to south. Billinudgel was compared to both the Split Solitary island and Charlesworth Bay due to their proximity. Bold values indicate a p-value < 0.005 (after Bonferroni correction).

|  | ADEGENET (n.sim = 999) | | | |
| --- | --- | --- | --- | --- |
|  | ∆He (=2-1) | | p-value two-sided | |
| Population comparison (1 - 2) | Neutral | Adaptive | Neutral | Adaptive |
| Moreton Is. - Billinudgel | 0.064 | 0.065 | **0.001** | **0.001** |
| Billinudgel - Split Solitary Is. | -0.003 | -0.014 | **0.001** | **0.001** |
| Billinudgel - Charlesworth Bay | -0.002 | -0.063 | **0.001** | 0.393 |
| Split Solitary Is. - Charlesworth Bay | 0.001 | 0.006 | **0.001** | **0.001** |
| Charlesworth Bay - Port Macquarie | 0.002 | 0.006 | **0.001** | **0.002** |
| Port Macquarie - Port Stephens | 0.01 | 0.000 | 0.025 | 0.457 |
| Port Stephens - Shellharbour | 0.029 | 0.006 | **0.001** | **0.001** |
| Shellharbour - Eden | 0.063 | -0.027 | **0.001** | **0.001** |
| Eden - Bicheno | -0.085 | -0.06 | **0.001** | **0.001** |
| Bicheno - Blackmans Bay | 0.014 | 0.006 | 0.039 | **0.004** |

**
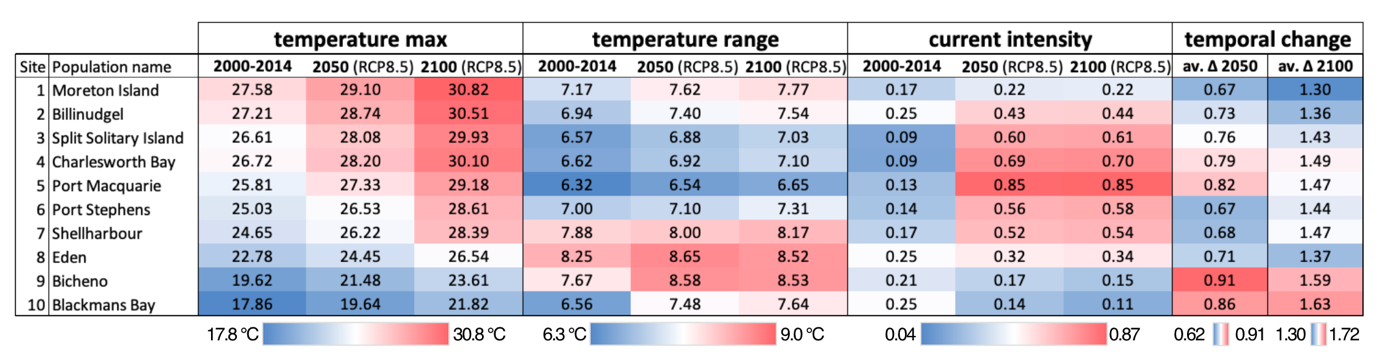
Table S5:** Extracted environmental predictor values for every site (1-10). Environmental predictors are temperature maximum (°C), temperature range (°C) and current intensity (m/s) at the mean bottom. Data was extracted from Bio-Oracle both for contemporary data (2000-2014) and for climatic predictions based on the RCP scenario 8.5 (2050 and 2100). The last two columns under ‘temporal change’ reflect w. Colour scale is provided under each category with the min. and max. values.

**Table S6:** Annotation for *E. radiata* gene models containing candidate SNPs. Hypothetical and conserved unknown proteins are not included. We provide SNP ID, variant type (intron= I, missense =M, synonymous = S), GenBank accession numbers, BLASTX E-value, annotation, molecular or biological GO terms and functions as described in literature.


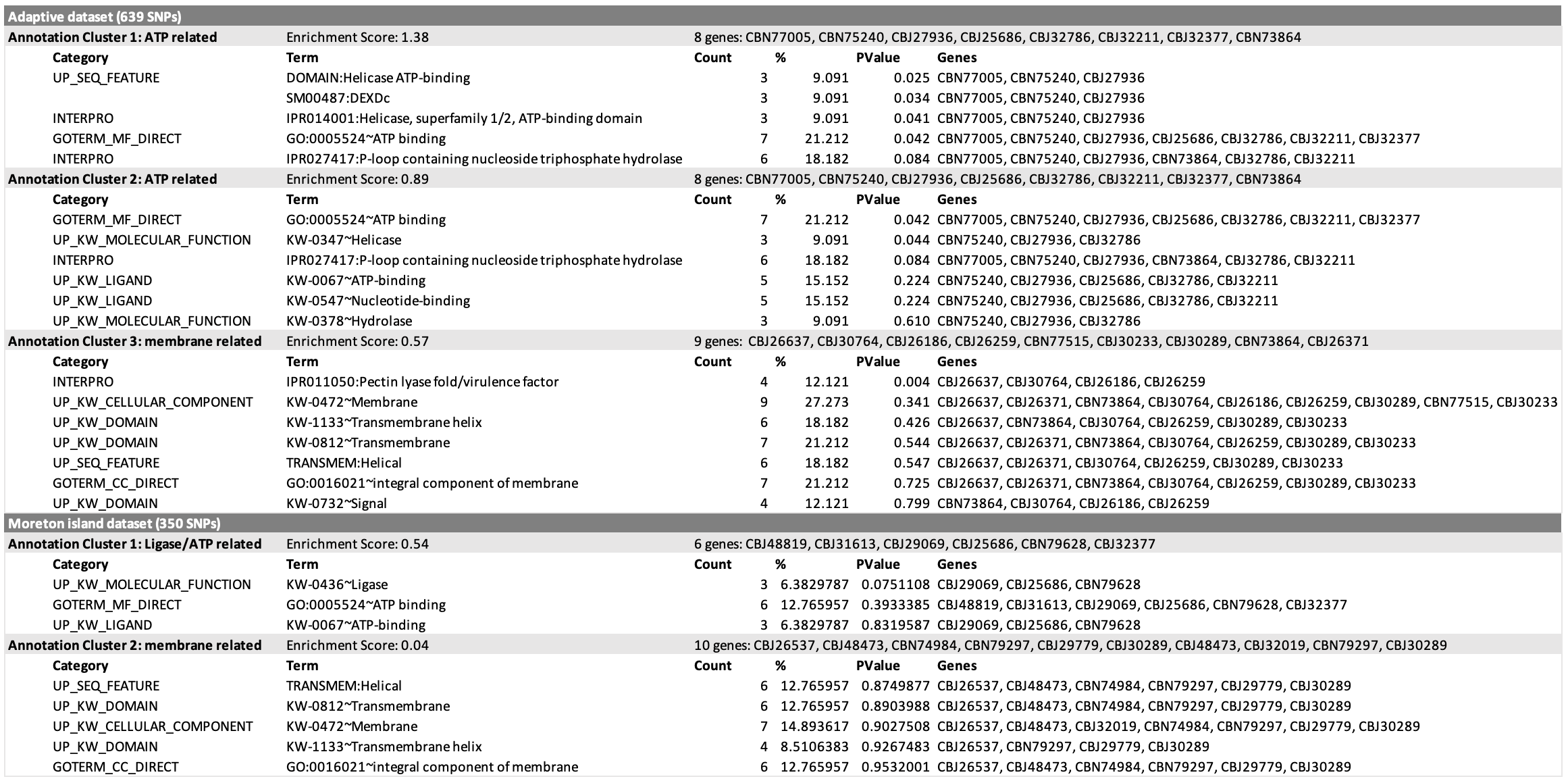


**Table S7:** Full list of successful Blast with a known gene function.

| Locus | ID | Gene Name |
| --- | --- | --- |
| Putative adaptive loci | |  |
| CLocus_327594 | CBJ32786 | ATP-dependent DNA helicase |
| CLocus_104174 | CBJ31291 | AVL9/DENND6 domain-containing protein |
| CLocus_163305 | CBJ26637 | Adhesin-like protein |
| CLocus_90138 | CBJ30233 | Alkane 1-monooxygenase |
| CLocus_170379 | CBJ32211 | Axonemal 1-beta dynein heavy chain dynein heavy chain |
| CLocus_94920 | CBN75052 | C2H2 zinc finger protein |
| CLocus_33483 | CBJ33082 | C2H2 zinc finger protein |
| CLocus_19463 | CBJ27636 | CCHC-type domain-containing protein |
| CLocus_54161 | CBJ27936 | Chromodomain-helicase-DNA-binding protein 8 |
| CLocus_59919 | CBN77515 | Conserved oligomeric Golgi complex subunit 2 |
| CLocus_101469 | CBN75240 | DEAD box helicase |
| CLocus_134926 | CBJ25686 | DNA ligase IV |
| CLocus_11328 | CBN73864 | Esi_0007_0185 |
| CLocus_40280 | CBJ26583 | Esi_0035_0072 |
| CLocus_12332 | CBN78136 | Esi_0100_0037 |
| CLocus_40732 | CBJ30289 | Esi_0184_0036 |
| CLocus_93897 | CBJ32377 | Esi_0334_0012 |
| CLocus_376021 | CBJ26259 | Extracellular nuclease |
| CLocus_45996 | CBN77005 | Helicase ATP-binding domain-containing protein |
| CLocus_59140 | CBN74642 | K Homology domain-containing protein |
| CLocus_124087 | CBN73823 | Methyltransferase type 12 |
| CLocus_134604 | CBN79585 | Myb-like DNA-binding domain containing protein |
| CLocus_7946 | CBJ32535 | NADAR domain-containing protein |
| CLocus_89812 | CBJ28025 | P/Homo B domain-containing protein |
| CLocus_62762 | CBJ30764 | Polymorphic membrane protein |
| CLocus_52260 | CBJ26186 | Polymorphic outer membrane protein |
| CLocus_115081 | CBJ31980 | Rieske (2Fe-2S) region |
| CLocus_8715 | CBJ25760 | Selenoprotein T (SELT) |
| CLocus_30264 | CBJ26371 | Similar to Probable phospholipid-transporting ATPase ID (ATPase class I type 8B member 2) |
| CLocus_1017932 | CBJ29448 | Splicing factor Cactin |
| CLocus_1761637 | CBN77525 | UDP-sugar pyrophosphorylase |
| Moreton island |  |  |
| CLocus_41982 | CBJ30979 | Alb3 homolog, thylakoidal inner membrane insertase |
| CLocus_35812 | CBJ27936 | Chromodomain-helicase-DNA-binding protein 8 |
| CLocus_130161 | CBJ30475 | Dynein heavy chain (DYHC11) |
| CLocus_116208 | CBJ29373 | EsV-1-7 |
| CLocus_14929 | CBJ29569 | Esi_0153_0026 |
| CLocus_14783 | CBJ32913 | Esi_0391_0017 |
| CLocus_10329 | CBJ30073 | EstExt_Genewise1.C_60040 |
| CLocus_134324 | CBJ31988 | Kinesin motor domain-containing protein(Esi_0298_0023) |
| CLocus_71662 | CBN79142 | Pentacotripeptide-repeat region of PRORP domain-containing protein(Esi_0010_0009) |
| CLocus_77510 | CBJ32323 | PhoD-like phosphatase metallophosphatase domain-containing protein(Esi_0330_0004) |
| CLocus_100953 | CBJ29835 | Probable extracellular nuclease |
| CLocus_1523428 | CBJ26156 | Putative katanin like protein |
| CLocus_88245 | CBJ27456 | RTW, Ras superfamily GTPase |
| CLocus_84590 | CBN77905 | SAM domain-containing protein |
| CLocus_56718 | CBN79477 | Sulfotransferase domain-containing protein |
